# Redox response feature and mechanism of Arf1 and their implications for those of Ras and Rho GTPases

**DOI:** 10.1016/j.jbc.2025.110269

**Published:** 2025-05-21

**Authors:** Hope Elizabeth Johnson, Emilynn Leigh Banks, Ostin Samuel Ozuna, Jongyun Heo

**Affiliations:** Department of Chemistry and Biochemistry, The University of Texas at Arlington, Arlington, Texas, USA

**Keywords:** Arf, redox, nitric oxide, superoxide, thiyl radical, guanine nucleotide radical

## Abstract

The small GTPases ADP-ribosylation factor (Arf), Ras, and Rho cycle between their active GTP-bound and inactive GDP-bound forms. Their regulatory proteins or inorganic redox agents regulate this cycle, which in turn regulates various important cell signals. Unlike regulatory protein-based regulation, redox-mediated regulation that occurs through the redox response of small GTPases to a redox agent is feasible only when the small GTPases are redox sensitive. The known redox-sensitive small GTPases including Ras and Rho have the reactive Cys in their unique redox motif. This study is the first to show the redox-response feature of Arf1 linked to a novel redox-sensitive Cys in the Arf-specific redox motif and its importance for cell functions. The Arf1 redox motif is currently the simplest form as it lacks the additional redox components found in Ras and Rho. The study also identifies critical radical intermediates implicated in the Arf1 redox response, along with the production of chemically modified nucleotides such as a GDP adduct. These results suggest the most elementary radical action-based mechanism for the Arf1 redox response. Although the presence of the radical intermediates was not reported, they were also suggested for the Ras and Rho redox response. Thus, the previously unknown mechanistic aspects of the Ras and Rho redox response are clarified by comparing them with those of Arf1.

ADP-ribosylation factor (Arf) is a small GTPase that belongs to the Arf family, whereas Ras and Rac are members of the Ras and Rho families of small GTPases, respectively (1, 2, 3). These small GTPases cycle between the active GTP- and inactive GDP-bound forms (4, 5, 6). The Arf GTPase activity cycle controls membrane trafficking and cellular organelle structures critical for cell growth and motility (7, 8, 9, 10, 11, 12, 13, 14, 15, 16, 17, 18). The activity cycles of Ras and Rho GTPases regulate cellular signaling transductions essential for cell survival, such as cell proliferation and organization (19, 20, 21, 22, 23, 24). The activity cycles of these small GTPases are regulated by their specific regulatory proteins, guanine nucleotide exchange factors (GEFs), and GTPase activating proteins (GAPs). GEFs produce active small GTPase by facilitating the exchange of bound GDP with cellularly abundant GTP to form the GTP-bound form (4, 5, 6, 25). GAPs counteract the function of GEFs by facilitating the hydrolysis of bound GTP to GDP, resulting in the inactive GDP-bound GTPase (4, 5, 6, 25).

Some biologically relevant redox agents include nitric oxide (^•^NO), nitrogen dioxide (^•^NO_2_), superoxide (O_2_^•−^), hydrogen peroxide (H_2_O_2_), and peroxynitrite (ONOO^−^) (5, 26, 27). Nitric oxide synthase (NOS) produces ^•^NO in cells (28, 29). NOS includes several isoforms such as neuronal NOS (nNOS) (30). ^•^NO exposure to O_2_ produces various forms of ^•^NO oxidation products such as ^•^NO_2_ and nitrogen trioxide (N_2_O_3_) (5, 31, 32, 33). N_2_O_3_ can be reversibly decomposed into ^•^NO and ^•^NO_2_ (5). Several sources of O_2_^•−^ are found in cells. NADPH oxidase (NOX) produces O_2_^•−^ (34, 35). Several isoforms of NOX such as NOX1 and NOX2 are known. Mitochondrial respiration is also known to produce O_2_^•−^ (36, 37). Superoxide dismutase (SOD) converts O_2_^•−^ into H_2_O_2_ (38, 39). Dual oxidase produces H_2_O_2_ (34, 40, 41). The reaction of ^•^NO with O_2_^•−^ produces the protonated form of peroxynitrite (ONOOH), with a p*K*_a_ of 6.8 (42). Thus, at the biologically relevant pH (6.5–8.5), it can also exist in the deprotonated form (ONOO^−^). Among these cell-based redox agents, ^•^NO, ^•^NO_2_, and O_2_^•−^ induce the redox response of a select few small GTPases, which are referred to as redox sensitive (43, 44, 45, 46, 47). The redox response of the redox-sensitive small GTPase mimics the GEF action because it also promotes the dissociation of bound GDP from small GTPases, allowing the binding of the cellularly abundant free GTP to produce active GTP-bound small GTPases (5). Thus, in the case of the redox-sensitive small GTPases, their activity control cycles also involve their redox responses and the action of GEFs.

The redox-sensitive features of the select small GTPases are due to the presence of the reactive Cys conserved in their specific redox motifs. H, K, and NRas of the Ras family GTPases possess the single-module redox NKCD motif (Fig. 1*A*) (48, 49). RhoA, RhoC, and Cdc42, which belong to Rho family GTPases, possess the redox GX_4_GK(S/T)C/ECS motif which is comprised of the GX_4_GK(S/T)C and ECS modules (Fig. 1*B*) (46, 50, 51). Depending on the local protein environment and the pH exposure, the redox-sensitive Cys sidechain in these motifs presents in the thiolate (Cys-S^−^) and/or sulfhydryl (Cys-SH) form. In any case, the reactive Cys side chain in these motifs is located close to the bound guanine nucleotide (GDP or GTP), which also interacts perpendicularly with Phe28 (Ras numbering, the perpendicular Phe) (Fig. 1) (47, 52).

**Figure 1:**
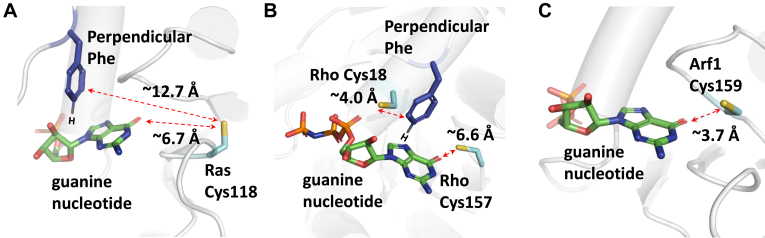
**Comparison of the spatial orientation between the lone Cys of Arf1 and the reactive Cys residues of Ras and Rho with the bound nucleotide base**. The local structures of the reactive Cys residue of Ras (PDB 1Q21) (*A*), the reactive Cys residues of Rho GX_4_GK(S/T)C/ECS motif (PDB 1MH1) (*B*), and the Arf1 lone Cys (PDB 6PTA) (*C*) the adjacent to the bound nucleotide are shown. These figures were generated using PyMol. Arf, ADP-ribosylation factor; PDB, Protein Data Bank.

The cellular roles of the redox-dependent Ras and Rho GTPases, each with a unique redox motif, have been previously reported. The redox-sensitive Ras is populated in its active state by the ^•^NO produced by nNOS in neuronal cells, which in turn stimulates Ras signaling cascades for neuronal functions (53). Our recent study also shows that redox-sensitive Rac becomes populated in its active form by O_2_^•−^ produced by NOX2, which then feedforward activates NOX2, resulting in Rac and NOX2 autoactivation (51). However, in contrast to Ras and Rho GTPases, the presence of the redox motif in Arf GTPases and their related redox response features has not been investigated. Therefore, the function of Arf in cells has uncertain redox dependencies.

Two possible yet distinctive redox response molecular mechanisms exist for Ras with NKCD and Rho with GX_4_GK(S/T)C/ECS motifs (Fig. 2). The radical action-based mechanism is based on the finding that the perpendicular Phe plays a part in the release of a chemically modified form of guanine nucleotide (nucleotide adduct, *e*.*g*., GDP or GTP adduct) (Fig. 2*A* and Supporting Information Fig. S1*A*) (47, 54). In this mechanism, ^•^NO_2_ or O_2_^•−^, but not ^•^NO, reacts with the redox-sensitive Cys-S^−^ to produce the reactive Cys thiyl radical (Cys-S^•^) of Ras NKCD motif. Through the perpendicular Phe, the reactive Cys-S^•^ then oxidizes the bound guanine nucleotide, producing the bound guanine radical cation (G^•+^) nucleotide. The perpendicular Phe in this step is suggested to function as a redox conduit between the reactive Cys and the bound guanine nucleotide. The bound G^•+^ nucleotide tautomerizes to the guanine radical (G^•^) nucleotide, which disrupts its hydrogen-bonding interactions with Ras and Rho, releasing the G^•^ nucleotide from them. The free G^•^ nucleotide is quenched by another redox agent, producing a chemically altered form of guanine nucleotide. This mechanism was then extended to the redox response of the Rho GX_4_GK(S/T)C/ECS motif because it likewise requires the action of the perpendicular Phe and releases a chemically modified form of guanine nucleotide (46, 50). Still, verification of the presence of these radical intermediates and the role of the perpendicular Phe for its redox-conduit function in the redox response of these GTPases remain unclarified. The thiol modification-based mechanism is based upon the finding of the formation of the S-nitrosylated reactive Cys (Cys-SNO) in the Ras redox response (Fig. 2*B* and Supporting Information Fig. S1*B*) (48). In this mechanism, it is suggested that the formation of Ras Cys-SNO in the Ras redox response induces a conformational change in the local structure of the guanine nucleotide-binding site of Ras which perturbs Ras nucleotide-binding interactions and subsequently results in the release of unmodified guanine nucleotide (*e*.*g*., GDP or GTP) from Ras. The same mechanisms that have been suggested for the production of free Cys-SNO may also be responsible for the formation of Ras Cys-SNO. The free Cys-SNO was suggested to be produced by the direct reaction of free Cys-SH with ^•^NO under anaerobic conditions in the presence of an electron acceptor such as NAD^+^ (Fig. 2B1) (55). It was also proposed that the free Cys-SNO can be formed by the reaction of free Cys-SH with a nitrosonium ion (NO^+^), and NO^+^ can be produced by the transition metal-mediated oxidation of ^•^NO (Fig. 2B2) (56). In any case, a key challenge of this thiol modification-based mechanism is explaining how the Ras redox response released the nucleotide adduct instead of the unmodified guanine nucleotide. It is also uncertain whether the suggested reaction mechanisms of the NAD^+^- and transition metal-mediated free Cys-SNO formation (Fig. 2B1 and 2B2) can be applied to the production of Ras Cys-SNO. This is because ^•^NO does not react with the reactive Cys side chain of Ras under anaerobic conditions, regardless of the presence of an electron acceptor or transition metals (33).

**Figure 2:**
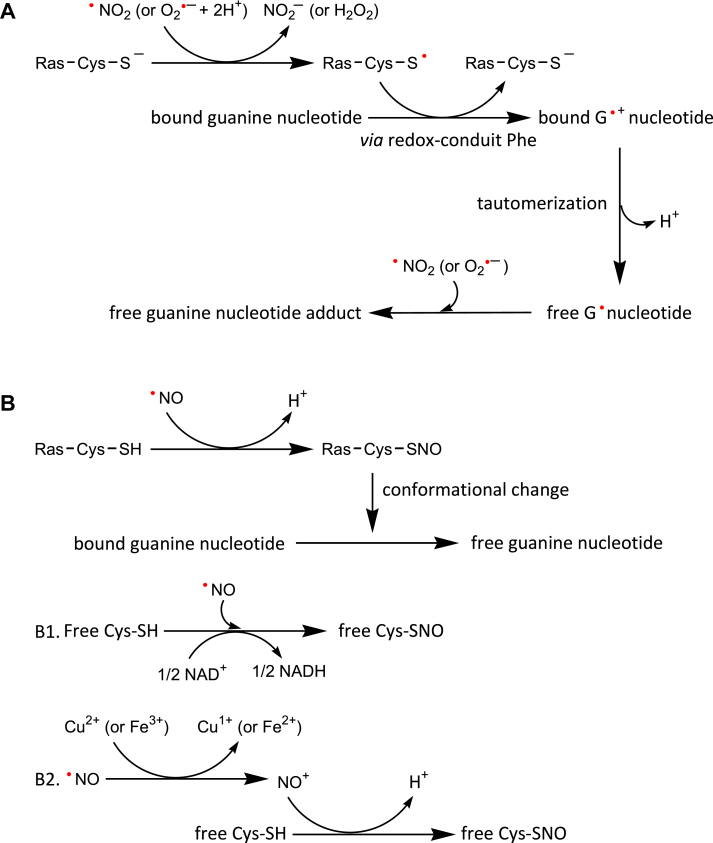
**Proposed reaction schemes for the Ras redox response.** The scheme for the radical action-based Ras redox response that releases the chemically altered nucleotide adduct (*A*) is shown. The scheme for the thiol modification-based Ras redox response that releases the unmodified nucleotide (*B*) is also shown. The proposed reaction schemes for free Cys-SNO formation in the presence of an oxidant NAD^+^ (*B1*) (55) and transition metals (*B2*) (56) are also shown. It is worth noting that the free Cys-SH and ^•^NO are suggested to react directly, yielding the free Cys-SNO and a hydrogen radical (H^•^). Although one-electron reduction of NAD^+^ to produce NADH is impossible, the stoichiometry of the reaction of the free Cys-SH with ^•^NO and NAD^+^ to produce the free Cys-SNO and NADH is set at 1:1/2 (*B1*). Detailed mechanistic steps described for the schemes *A* and *B* are shown in Figure S1.

This study first shows the novel Arf1 redox response features associated with finding a new reactive Cys (Cys159 Arf1 numbering) in a unique redox motif (Fig. 1*C*). Unlike Ras and Rho GTPases, Arf1 has only one reactive Cys, therefore it is referred to as a lone Cys. Intriguingly, Arf1 inherently lacks Phe, which corresponds to the perpendicular redox-conduit Phe in Ras and Rho (Fig. 1). This study also identifies critical redox reaction intermediates in the unique Arf1 redox response. The study further shows the biological importance of the Arf1 redox response in cell function. Taken together, this study presents a molecular mechanism that underlies the Arf1 redox response associated with the unique Arf1 reactive Cys. A comparison of the proposed Arf1 redox response mechanism to those of Ras and Rho elucidates previously unclear mechanistic aspects of the Ras and Rho redox responses. One of these aspects is whether the redox-conduit role of the perpendicular Phe is necessary for Ras and Rho redox responses. Another aspect is whether the proposed redox reaction intermediates (reactive Cys-S^•^ and bound G^•+^ or G^•^ nucleotide) or the thiol modification have a role in the redox responses of Ras and Rho. These aspects help to determine whether Ras and Rho redox responses are mediated by radical action or thiol modifications.

## Results

### Kinetics of the Arf1 redox response with various redox agents

To examine whether wt Arf1 is redox-sensitive, the kinetic effects of biologically relevant redox agents on the guanine nucleotide exchange of wt Arf1 were examined under various experimental conditions.

**Figure 3:**
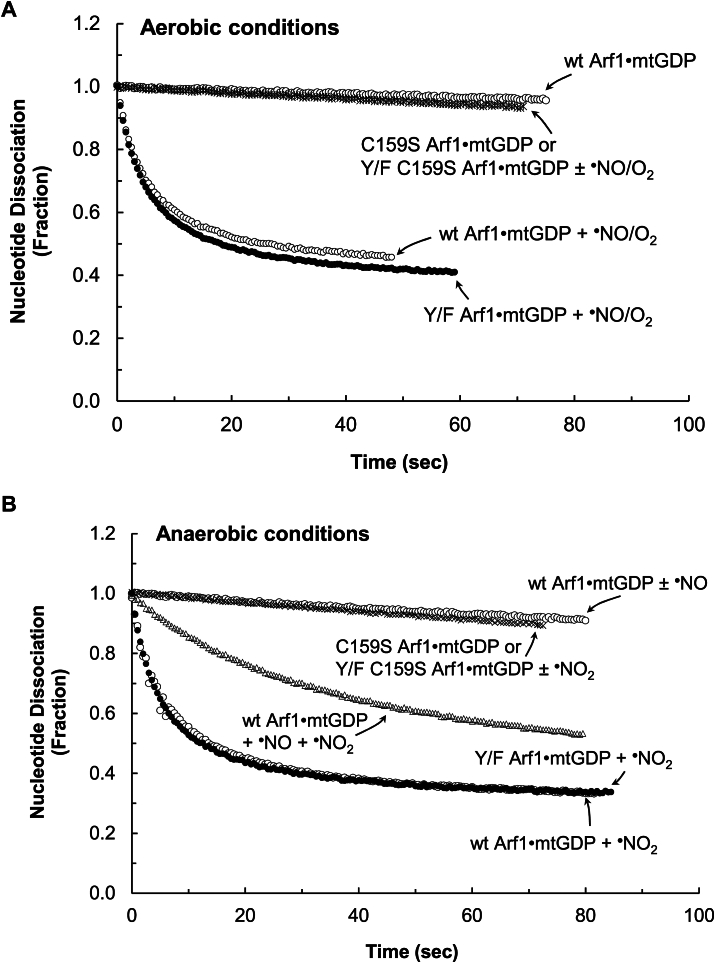

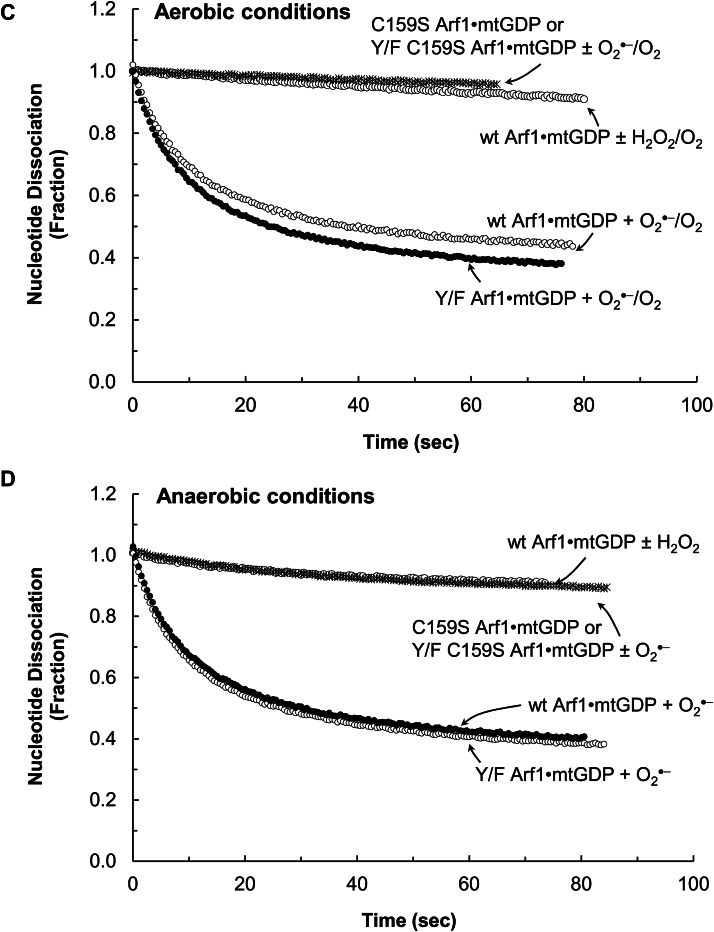

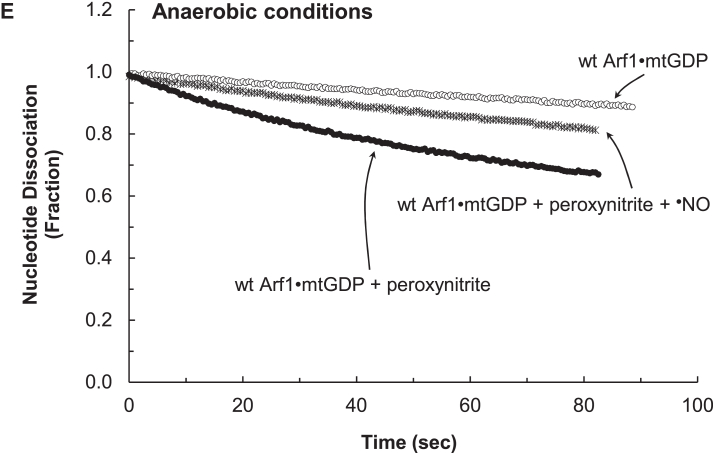
**Examination of the Arf1 GDP redox response with various redox agents under aerobic and anaerobic conditions**. Fluorescent mant-based nucleotide dissociations from wt and C159S Arf1 by ^•^NO under aerobic conditions (*A*), ^•^NO_2_ and/or ^•^NO under anaerobic conditions (*B*), O_2_^•−^ or H_2_O_2_ under aerobic conditions (*C*), O_2_^•−^ or H_2_O_2_ under anaerobic conditions (*D*), and peroxynitrite under anaerobic conditions (*E*) are shown. Assay solutions with various redox agent concentrations in a sealed assay cuvette and the mant GDP-loaded Arf1 protein stock in a sealed vial (10 μM) were produced anaerobically, as described in the experimental procedures section. For the aerobic assays, as described in the experimental procedures section, atmospheric O_2_ was introduced using a syringe before the initiation of the assay. Otherwise, the atmospheric O_2_ injection was skipped. The assay was begun by injecting anaerobic mtGDP-bound Arf1 (final concentration 0.1 μM) using a N_2_-flushed syringe into a sealed assay cuvette containing aerobic (when O_2_ was injected) or anaerobic assay solution with a specific redox agent at various concentrations (1–50 μM), as noted in the experimental procedures section. The changes in mant fluorescence in each set of experiments were fractionated by dividing them with their maximal fluorescence values, and then a simple exponential decay function was fit to the data to obtain the first-order rate constant (*k*) of mtGDP dissociation from Arf1. To avoid confusion, only the results that showed the maximal *k* values of mtGDP dissociation from Arf1 in each set of experiments at specific concentrations of the redox agent were shown. The *k* values of mtGDP dissociation from wt and Y/F Arf1 with ^•^NO (∼45 μM) under aerobic conditions and with ^•^NO_2_ (∼10 μM) under anaerobic conditions were estimated to be 0.129 ± 0.022 s^−1^. The *k* value of mtGDP dissociation from wt Arf1 with ^•^NO_2_ (∼10 μM) plus ^•^NO (∼10 μM) under anaerobic conditions was 0.05 ± 0.007 s^−1^. The *k* value of mtGDP dissociation from wt and Y/F Arf1 with O_2_^•−^ (∼30 μM) under aerobic and anaerobic conditions was 0.087 ± 0.014 s^−1^. The *k* value of mtGDP dissociation from wt Arf1 with peroxynitrite (∼50 μM) under anaerobic conditions was 0.02 ± 0.002 s^−1^. All other reactions, including mtGDP dissociation from wt and Y/F Arf1 with H_2_O_2_ (∼50 μM) under aerobic and anaerobic conditions as well as with peroxynitrite (∼50 μM) plus ^•^NO (∼100 μM) under anaerobic conditions, had negligible *k* values (<0.05 s^−1^). *R*^2^ values for these fittings were > 0.9995. Arf, ADP-ribosylation factor.

Figure 3*A* shows that ^•^NO, under the aerobic conditions, could promote GDP dissociation from wt Arf1. The maximal rate of the ^•^NO-mediated wt Arf1 GDP dissociation under the aerobic conditions was ∼0.13 s^−1^ at ∼45 μM ^•^NO. Because ^•^NO can react with O_2_ in ambient air to produce higher oxides such as ^•^NO_2_ and N_2_O_3_, wt Arf1 treated with ^•^NO under aerobic conditions may have been exposed to a mixture of unreacted ^•^NO, ^•^NO_2_, and N_2_O_3_. Therefore, the reaction species responsible for GDP dissociation from wt Arf1 when treated with ^•^NO under aerobic conditions remains unclear. To identify the redox agent that promotes wt Arf1 GDP dissociation under aerobic conditions, a wt Arf1 GDP dissociation assay was performed under strict anaerobic conditions using ^•^NO and/or ^•^NO_2_. Because of these conditions, the treatment ^•^NO or ^•^NO_2_ was prevented from reacting with the ambient O_2_. Figure 3*B* shows that ^•^NO alone, under strict anaerobic conditions, was ineffective in increasing wt Arf1 GDP dissociation. However, Figure 3*B* also shows that, under strict anaerobic conditions, ^•^NO_2_ could increase GDP dissociation from wt Arf1. Under strict anaerobic conditions, the maximal rate of ^•^NO_2_-mediated wt Arf1 GDP dissociation was ∼0.13 s^−1^ at ∼10 μM ^•^NO_2_ (Fig. 3*B*), which was similar to that of ^•^NO-mediated wt Arf1 GDP dissociation under aerobic conditions at ∼45 μM ^•^NO (Fig. 3*A*). The results suggest that ^•^NO_2_, but not ^•^NO, could enhance GDP dissociation from wt Arf1. Figure 3*B* further shows that the rate of wt Arf1 GDP dissociation was reduced when ^•^NO was introduced to the assay system in addition to ^•^NO_2_ (1 ^•^NO mol/1 ^•^NO_2_ mol) under the strict anaerobic conditions. In the absence of O_2_, ^•^NO_2_ reacts with ^•^NO to produce N_2_O_3_. Thus, the addition of ^•^NO to the ^•^NO_2_-containing assay system reduced ^•^NO_2_ concentrations while raising N_2_O_3_ concentrations. If the N_2_O_3_ produced could increase wt Arf1 GDP dissociation, this rate decrease would not have occurred. The observed rate decrease could be related to a decrease in ^•^NO_2_ concentrations, which promoted GDP dissociation from wt Arf1. Thus, N_2_O_3_ apparently cannot enhance GDP dissociation from wt Arf1. Overall, the results and analyses suggest that ^•^NO_2_, rather than ^•^NO or N_2_O_3_, is the actual redox agent that promotes GDP dissociation from wt Arf1 when treated with ^•^NO under aerobic conditions.

Figure 3*C* shows that O_2_^•−^ can promote GDP dissociation from wt Arf1 in aerobic conditions. The maximum rate of O_2_^•−^-mediated wt Arf1 GDP dissociation under aerobic conditions was ∼0.09 s^−1^ at ∼30 μM O_2_^•−^. The maximal rate of O_2_^•−^-mediated wt Arf1 GDP dissociation remained unchanged under strict anaerobic conditions (Fig. 3*D*). This constancy is consistent with the fact that O_2_^•−^ does not react with O_2_ to produce other oxygen forms. The results suggest that, unlike ^•^NO, O_2_^•−^ *per se* but not its reaction product with O_2_ promotes wt Arf1 GDP dissociation under aerobic conditions. Figure 3, *C* and *D* show that Arf1 GDP dissociation by H_2_O_2_ (∼30 μM) was not observed under aerobic or anaerobic conditions. Overall, these results suggest that O_2_^•−^, but not H_2_O_2_, promotes GDP dissociation from wt Arf1, regardless of the presence of O_2_.

Figure 3*E* shows that peroxynitrite can also promote GDP dissociation from wt Arf1 in anaerobic conditions. The maximum rate of the peroxynitrite-mediated wt Arf1 GDP dissociation under the conditions was ∼0.02 s^−1^ at ∼50 μM ONOO^−^. This rate was much slower than that of the rate with ^•^NO_2_ or O_2_^•−^ under the identical experimental conditions. At the experimental pH (7.4), ∼85% of peroxynitrite was ONOO^−^, and ∼15% was ONOOH, according to the p*K*_a_ of peroxynitrite (42). Unlike ONOO^−^, ONOOH can be cleaved homolytically into hydroxyl radical (OH^•^) and ^•^NO_2_. The highly reactive OH^•^ (5) could react nonspecifically with a number of Arf1 protein residues. Therefore, the OH^•^ reaction in the assay system cannot be specifically attributed to the Arf1 redox response. ^•^NO_2_ was identified as the reactive species that promotes GDP dissociation from wt Arf1 (see above). If ^•^NO_2_ was the main active redox agent in the peroxynitrite-mediated wt Arf1 GDP dissociation, quenching it with ^•^NO to produce N_2_O_3_ could block it. Otherwise the active redox agent in the system can be either the uncleaved ONOO^−^ or ONOOH, or both. Figure 3*E* shows that, under the anaerobic conditions, the rate of wt Arf1 GDP dissociation was significantly reduced when ^•^NO was introduced to the assay system in addition to peroxynitrite (2 ^•^NO mol/1 peroxynitrite mol). The result supports the notion that ^•^NO_2_, a homolytic cleavage product of ONOOH, rather than ONOO^−^ *per se*, was responsible for the peroxynitrite-mediated wt Arf1 GDP dissociation. The observed slow peroxynitrite-mediated wt Arf1 GDP dissociation could be attributed to the slow rate of homolytic cleavage of ONOOH to yield OH^•^ and ^•^NO_2_ (27, 42).

In contrast to the case of the GDP dissociation from wt Arf1 by ^•^NO under aerobic conditions and ^•^NO_2_ under strict anaerobic conditions, neither ^•^NO under aerobic conditions nor ^•^NO_2_ under strict anaerobic conditions promoted the association of a fresh GDP to nucleotide-deficient wt Arf1 (not shown). O_2_^•−^ also failed to promote GDP association with nucleotide-deficient wt Arf1 under aerobic and anaerobic conditions (not shown). These results suggest that the wt Arf1 redox response by ^•^NO_2_ and O_2_^•−^ is unidirectional, promoting dissociation GDP from Arf1 but not its association.

The same redox kinetic results were observed when the GTP-bound wt Arf1 was used instead of the GDP-bound wt Arf1. For example, ^•^NO under aerobic conditions and ^•^NO_2_ under anaerobic conditions were capable of promoting GTP dissociation from wt Arf1 (not shown). The GTP dissociation from wt Arf1 was also enhanced by O_2_^•−^ but not H_2_O_2_ under aerobic and anaerobic conditions (not shown). Furthermore, as with GDP, none of these redox agents was capable of increasing GTP binding to the nucleotide-deficient wt Arf1 (not shown). As a result, the unidirectional action of these redox agents on wt Arf1 is not particular to the bound nucleotide form, either GDP or GTP, and thus is not specific to the activity status of wt Arf1. In cells, small GTPases, including Arf1, are mostly populated as their inactive GDP-bound forms. Thus, the unidirectional redox action on wt Arf1, which is primarily GDP-bound but not exclusively GDP-bound, results in the production of a nucleotide-deficient wt Arf1. However, in cells, GTP is more abundant than GDP (57). Thus, by mass action, GTP binds back to the formed nucleotide-deficient wt Arf1 to produce active GTP-bound Arf1 in cells. As a result, the overall outcome of the redox action is to populate the active GTP-bound Arf1.

### Identification of the target site of the Arf1 redox response

The reactive Cys side chain in the NKCD and GX4GK(S/T)C/ECS motifs are the target sites of ^•^NO_2_ or O_2_^•−^, which elicit the Ras and Rho redox responses, respectively (5). Intriguingly, the lone Cys of wt Arf1 is located near the bound guanine nucleotide, which is similar to the reactive Cys in the NKCD and GX_4_GK(S/T)C/ECS motifs (Fig. 1). Thus, the Arf1 lone Cys is a potential target for these redox agents to elicit the Arf1 redox response. To investigate this possibility, the redox response of the lone Cys mutant C159S Arf1 with ^•^NO_2_ and O_2_^•−^ under various conditions was examined. Arf1 also possesses several residues of Tyr (35, 58, 81, 154, and 167, Arf1 numbering). All of these Tyr are solvent exposed (Arf1 Protein Data Bank (PDB) 6PTA). In some cases, Tyr in proteins is a target of biologically relevant redox agents such as H_2_O_2_ (58, 59, 60, 61, 62). Thus, the Arf1 Tyr residues are potential targets of ^•^NO_2_ and O_2_^•−^. This Tyr-targeting redox action may also link to the Arf1 redox response. To examine this possibility, all of these Tyr residues of Arf1 were mutated into Phe to produce Y35F/Y58F/Y81F/Y154F/Y167F Arf1 (Y/F Arf1 mutant), and its redox response kinetic features with ^•^NO_2_ and O_2_^•−^ were also analyzed in this section under the various experimental conditions. Unlike the case of Tyr, Phe is known to not react with ^•^NO_2_ and O_2_^•−^. Thus, this mutant could lack the potential effects of the Tyr reactions with ^•^NO_2_ and O_2_^•−^ on the Arf1 redox response. The lone Cys mutation (C159S) was also introduced in the Tyr mutant to produce the Y/F C159S Arf1 mutant, which was used as a control for the redox response study of the Y/F Arf1 mutant.

Figure 3, *A* and *B* show that Arf1 GDP dissociation was not observed when C159S Arf1 was treated with ^•^NO under aerobic conditions or ^•^NO_2_ under strict anaerobic conditions. Furthermore, no GDP dissociation from C159S Arf1 with O_2_^•−^ was observed under aerobic and anaerobic conditions (Fig. 3, *C* and *D*). These results suggest that the lone Cys is redox-sensitive and thus is a target of ^•^NO_2_ or O_2_^•−^ to elicit the Arf1 redox response. Figure 3, *A*–*D* also show that ^•^NO under aerobic conditions, ^•^NO_2_ under anaerobic conditions, and O_2_^•−^ under aerobic and anaerobic conditions could promote GDP dissociation from Y/F Arf1 but not from the Y/F C159S Arf1 mutant. The results thus further suggest that only the lone Cys, but not the Tyr residues, of Arf1 is implicated in the Arf1 redox response.

### Determination of the mode of the mechanism of the Arf1 redox response

The Arf1 redox response is novel. Yet, the common denominator of the redox response of Arf1 with other proteins such as Ras and Rho GTPases is its dependency on the reactive Cys residues in their specific redox motifs. The reactive Cys-dependent Ras and Rho redox responses have been modeled using two distinct modes of mechanism: radical- and thiol modification-based mechanism. An essential hallmark of the radical- and thiol modification-based mechanisms is, respectively, the nucleotide adduct production and Cys-SNO formation. To examine whether any of these mechanisms apply to the Arf1 redox response associated with the redox-sensitive lone Cys, the potential nucleotide adduct production and lone Cys-SNO formation were examined by using electrospray ionization mass spectrometry (ESI-MS) analysis on the Arf1 redox response samples under various conditions.

**Figure 4:**
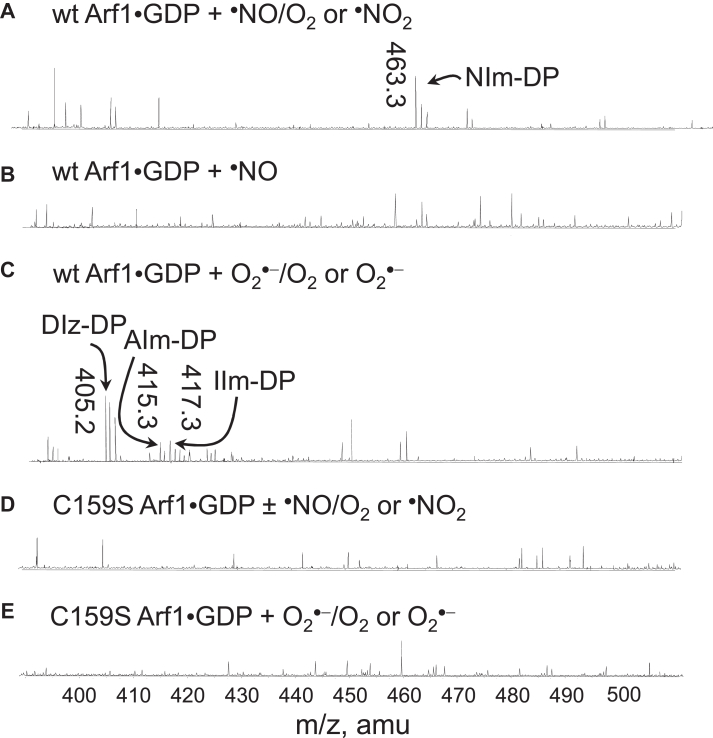
**Identification of nucleotide types released from Arf1 in the Arf1 redox response**. The masses of the nucleotides and their adducts released from the sample of GDP-bound wt Arf1 treated with ^•^NO under aerobic conditions or ^•^NO_2_ under anaerobic conditions (*A*), ^•^NO under anaerobic conditions (*B*), and O_2_^•−^ under aerobic and anaerobic conditions (*C*) are shown. The masses of the nucleotides and their adducts released from the sample of GDP-bound C159S Arf1 treated with ^•^NO under aerobic conditions or ^•^NO_2_ under anaerobic conditions (*D*) and O_2_^•−^ under aerobic and anaerobic conditions (*E*) are also shown. These mass analyses were performed using ESI-MS as described in the experimental procedures section. The peak at 493.3 Da is assigned to NIm-DP, the degradation product of the GDP-NO_2_ adduct. The peaks at 405.2, 415.3, and 417.3 Da. are assigned to 2,5-diamino-4H-imidazolone ribose diphosphate (DIz-DP), 5-amino-4O-imidazolone ribose diphosphate (AIm-DP), and 5-imino-4O-imidazolone ribose diphosphate (IIm-DP), respectively. Arf, ADP-ribosylation factor; ESI-MS, electrospray ionization mass spectrometry.

Figure 4*A* shows that the degradation product of the guanine nucleotide-NO_2_ adduct was detected whenever wt Arf1 GDP dissociation was promoted by ^•^NO under aerobic conditions or by ^•^NO_2_ under strict anaerobic conditions. The unmodified guanine nucleotide (*e*.*g*., GDP) was not detected in the sample of wt Arf1 treated with ^•^NO under aerobic conditions or ^•^NO_2_ under strict anaerobic conditions (Fig. 4*A*). Furthermore, only the guanine nucleotide-O_2_ adduct degradation products, but not the unmodified guanine nucleotide, were detected in the wt Arf1 sample treated with O_2_^•−^ under the aerobic and anaerobic conditions (Fig. 4*C*). These results suggest that the radical action-based mechanism for the Arf1 redox response that produces the nucleotide adduct probably applies to the Arf1 redox response. Moreover, Figure 4*B* shows that when wt Arf1 was treated with ^•^NO under anaerobic conditions, neither the nucleotide adduct degradation products nor the unmodified guanine nucleotide were found. Nothing was detected in the C159S Arf1 samples treated with O_2_^•−^ under aerobic and anaerobic conditions, ^•^NO_2_ under strict anaerobic conditions, and ^•^NO under aerobic conditions (Fig. 4, *D* and *E*). These results were not unexpected because neither the promotion of wt Arf1 GDP dissociation by ^•^NO under anaerobic conditions nor the promotion of C159S Arf1 GDP dissociation by ^•^NO, ^•^NO_2_, and O_2_^•−^ under aerobic and anaerobic conditions were observed (see above). The results further suggest that a nucleotide adduct, not an unmodified guanine nucleotide, is released when a redox agent promotes Arf1 GDP dissociation.

**Figure 5:**
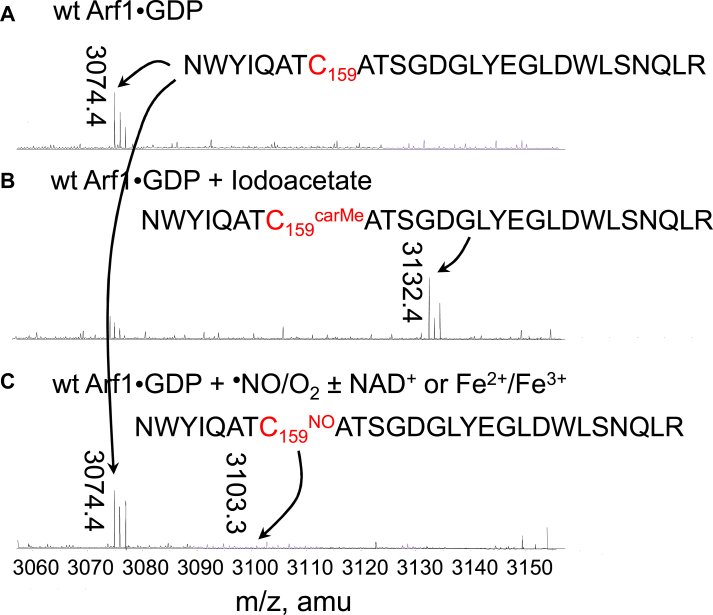
**Detection of the potential thiol modification of Arf1 in the Arf1 redox response**. The masses of the peptides from the trypsin-digested sample of GDP-bound wt Arf1 untreated (*A*), treated with iodoacetate (*B*), and treated with ^•^NO under aerobic conditions in the presence and absence of NAD ^+^ or Cu^2+^ (*C*) are shown. The peptide mass analyses were conducted using ESI-MS as described in the experimental procedures section. The masses at 3074.4 and 3132.4 Da are assigned to the peptide with the unmodified Cys159 (C_159_) and carboxymethylated Cys159 (C_159_^CarMe^), respectively, whereas the mass at 3103.3 Da is assigned to the peptide with S-nitrosylated Cys159 (C_159_^NO^). Arf, ADP-ribosylation factor.

Figure 5, *A* and *B* show that the lone Cys carboxymethylation occurs upon the treatment of wt Arf1 with iodoacetate. Figure 5*C*, however, shows that the lone Cys-SNO, the potential Arf1 lone Cys thiol modification product, was not detected in the wt Arf1 sample treated with ^•^NO under aerobic conditions. These results suggest that, although the lone Cys is susceptible to a chemical modification, it was unlikely to react with ^•^NO under aerobic conditions to produce the lone Cys-SNO. Notably, wt Arf1 treated with ^•^NO under aerobic conditions resulted in the release of the nucleotide adduct but not the unmodified guanine nucleotide (see the kinetic analysis section). It can be therefore suggested that the thiol modification-based mechanism proposed for Ras is unlikely to apply to the Arf1 redox response. Furthermore, there were two putative free Cys-SNO formation mechanisms that may be applicable for the Ras Cys-SNO formation (Fig. 2): the direct reaction of free Cys-SH with ^•^NO under anaerobic conditions in the presence of NAD^+^ (Fig. 2B1); and the reaction of free Cys-SH with NO^+^, where NO^+^ can be produced by the oxidation of ^•^NO mediated by transition metals (Fig. 2B2). However, no wt Arf1 redox response occurred when the wt Arf1 sample was treated with ^•^NO under anaerobic experimental conditions in the presence of NAD^+^ or transition metals (not shown). Arf1 lone Cys-SNO formation was also undetected in the wt Arf1 sample treated with ^•^NO under these experimental conditions (Fig. 5*C*). Thus, as with the thiol modification-based redox response mechanism proposed for Ras, the mode of free Cys thiol modification with NAD^+^ or transition metals lacks chemical precedent and thus was also inapplicable to Arf1 lone Cys thiol modification with respect to the redox response function of the Arf1 lone Cys.

### Occurrence of radical intermediates in the action of the lone Cys in the Arf1 redox response

The radical action-based mechanisms for Ras and Rho redox responses are proposed to involve subsequent radical intermediates, reactive Cys-S^•^, and the bound G^•+^ and free G^•^ nucleotides (5, 45, 46, 47). The lone Cys of Arf1 is equivalent to the reactive Cys that is the precursor of the reactive Cys-S^•^ in Ras and Rho. As with Ras and Rho, Arf1 also binds with a nucleotide such as GDP, which is the precursor of the bound G^•+^ and free G^•^ nucleotides, in its nucleotide binding site. Accordingly, when the radical action-based mechanism for the Arf1 redox response is applied, the radical intermediates, the lone Cys-S^•^, and the bound G^•+^ and free G^•^ nucleotides are also predicted to form. This prediction was examined using the time-resolved rapid freeze quench (RFQ) electron paramagnetic resonance (EPR) spectrometer on Arf1 samples treated with O_2_^•−^ or ^•^NO_2_ under strict anaerobic conditions. The kinetic study also shows that Tyr residues of Arf1 were not involved in the Arf1 redox response. Even so, although they play no role in the Arf1 redox response, these Arf1 Tyr residues can be a target of O_2_^•−^ or ^•^NO_2_ to produce their radicalized forms. In this form, their EPR signals can potentially overlap those of the potential lone Cys-S^•^ and the bound G^•+^ and free G^•^ nucleotides. In this case, the potential EPR signals associated with Arf1 Tyr residues must be identified to distinguish them from the EPR signals of the lone Cys-S^•^ and the bound G^•+^ and free G^•^ nucleotides. Therefore, the Arf1 Tyr mutants (Y/F and Y/F C159S Arf1) were also used for the RFQ EPR analyses as controls.

**Figure 6:**
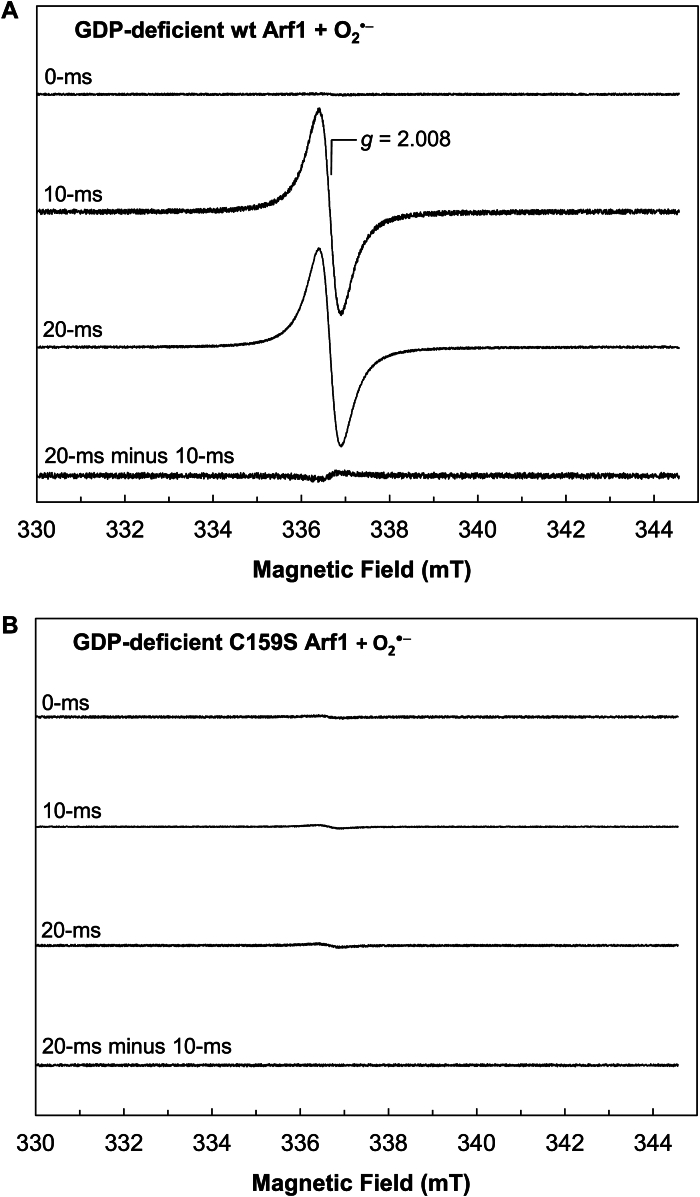

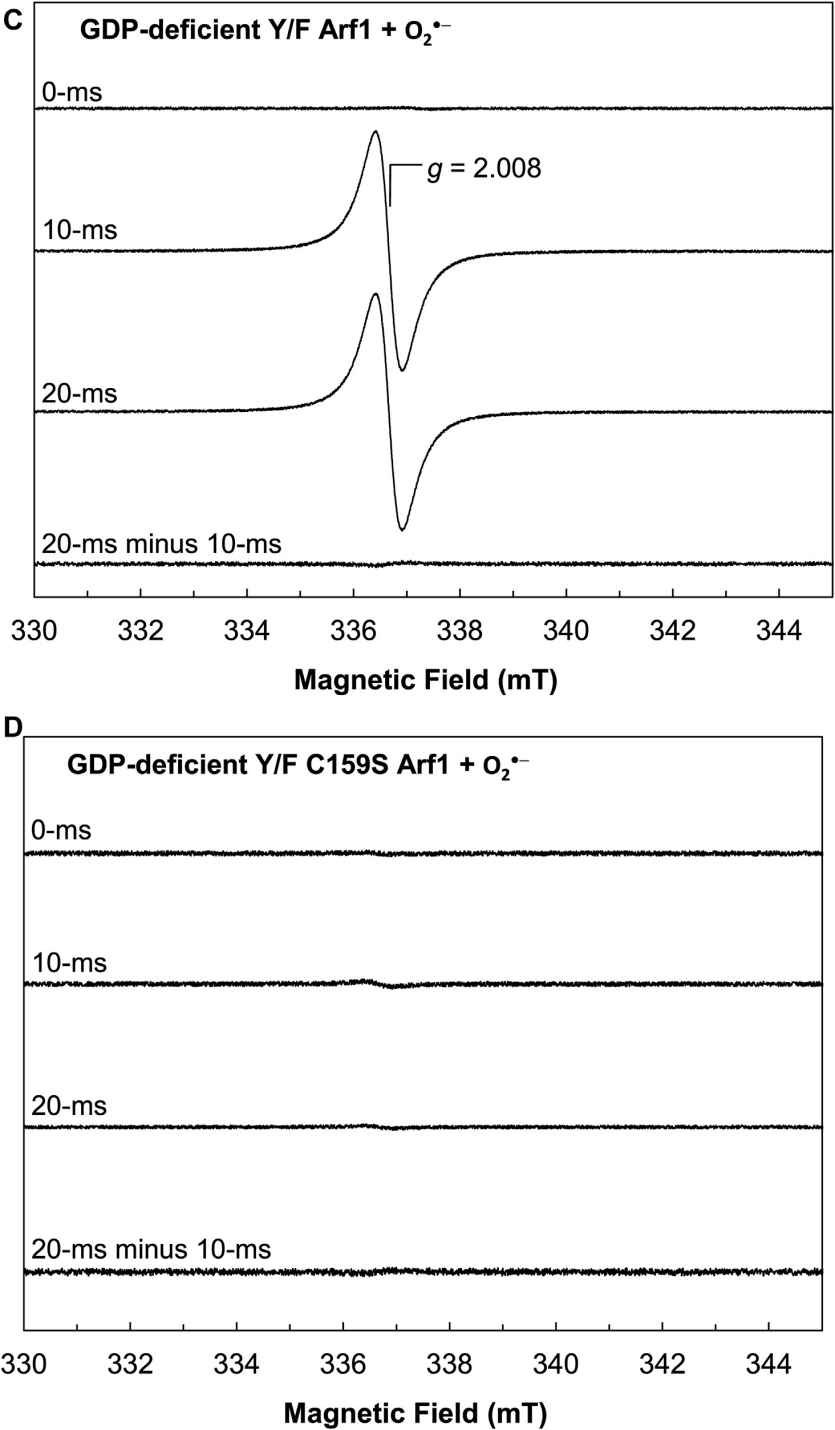

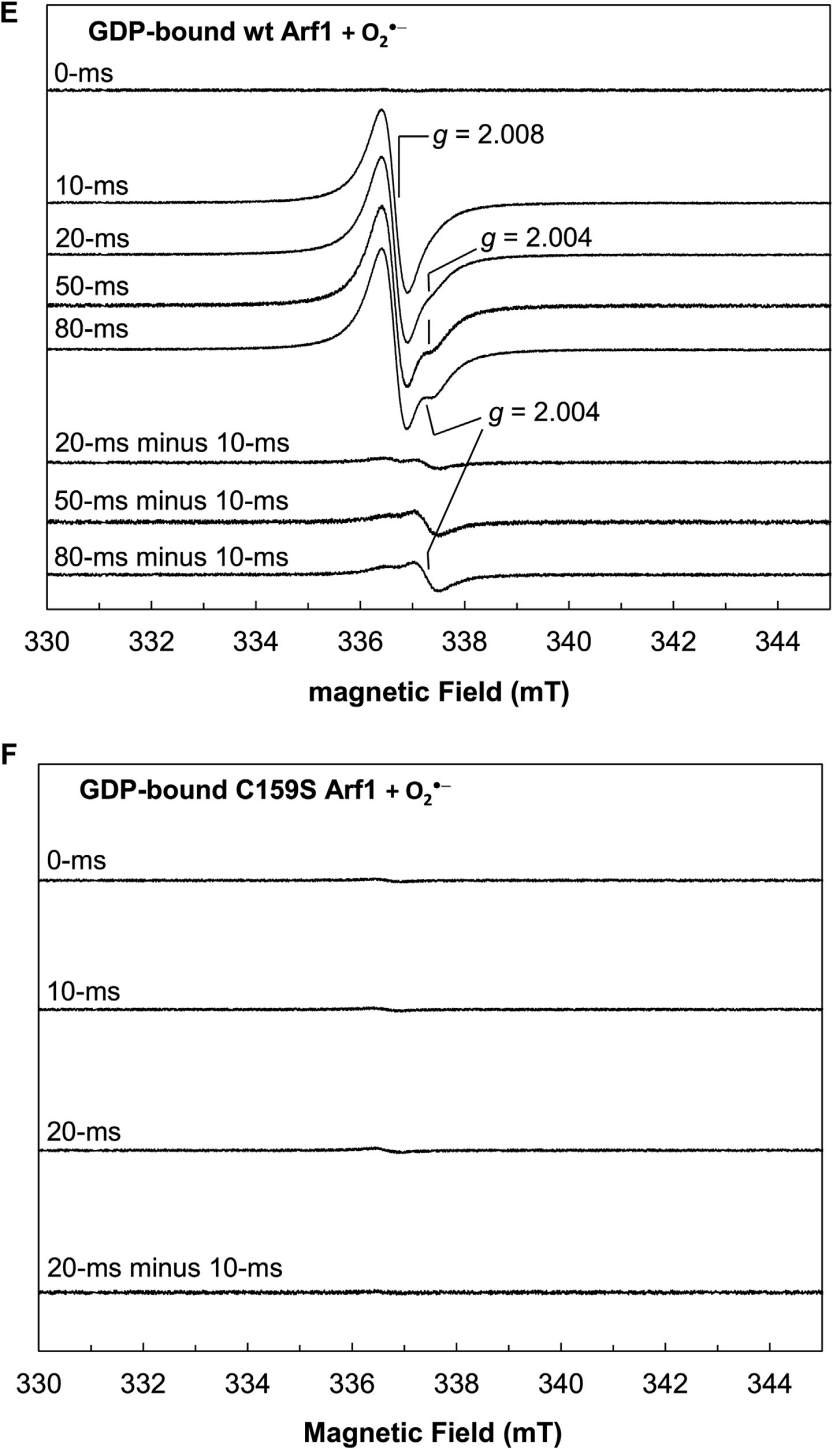
**Identification of the transient radicals formed in the Arf1 redox response.** The RFQ EPR spectra of the nucleotide-deficient form of wt, C159S, Y/F, and Y/F C159S Arf1 treated with O_2_^•−^ (*A*, *B*, *C*, and *D*, respectively) as well as the GDP-bound form of wt and C159S Arf1 treated with O_2_^•−^ (*E* and *F*, respectively) are shown. RFQ EPR analyses were performed as described in the experimental procedures section. The *g* value of 2.008 is assigned for the lone Cys-S^•^, whereas the deconvoluted *g* value of 2.004 is assigned for the bound G^•+^ and/or free G^•^ nucleotides. The EPR spectra were taken at 10 K. EPR conditions: microwave frequency = 9.42 GHz, microwave power = 1.0 mW, modulation amplitude = 10.5 G, and modulation frequency = 100 kHz. Arf, ADP-ribosylation factor; RFQ, rapid freeze quench; EPR, electron paramagnetic resonance.

Figure 6*A* shows that the nucleotide-deficient wt Arf1 RFQ sample at 10 ms exhibits EPR spectra with *g* = 2.008. The radical action-based mechanism is proposed to involve the lone Cys-S^•^ and bound G^•+^ and free G^•^ nucleotide intermediates during the wt Arf1 redox response. However, this nucleotide-deficient wt Arf1 RFQ sample also lacks the nucleotide, which is the precursor of the bound G^•+^ and free G^•^ nucleotides. Thus, the *g* = 2.008 EPR spectra of the nucleotide-deficient wt Arf1 RFQ sample at 10 ms cannot originate from the bound G^•+^ and free G^•^ nucleotides. Furthermore, Figure 6*B* shows that the EPR signal with *g* = 2.008 did not arise from the nucleotide-deficient C159S Arf1 RFQ sample at 10 ms. When combined, the origin of the *g* = 2.008 EPR spectra can be assigned to the lone Cys-S^•^. This assignment is consistent with the *g* value of the typical Cys-S^•^ in other systems (63, 64). The assignment is also essentially supported by the fact that the *g* = 2.008 EPR signal only arises from the RFQ sample of the nucleotide-deficient Y/F Arf1 mutant at 10 ms (Fig. 6*C*). This finding is obtained because, with the exception of the lone Cys, no other residues in the nucleotide-deficient Y/F Arf1 can directly react with O_2_^•−^ to generate radical species that give rise to the *g* = 2.008 EPR spectra under the given conditions. The assignment of the *g* = 2.008 EPR spectra to the lone Cys-S^•^ in the nucleotide-deficient Y/F Arf1 mutant was further validated by the absence of these spectra at 10 ms for the nucleotide-deficient Y/F C159S Arf RFQ sample (Fig. 6*D*), which lacks all potential redox-sensitive residues, including the lone Cys, that can react with O_2_^•−^ or ^•^NO_2_ to form the lone Cys-S^•^.

Figure 6*E* shows that the wt Arf1 RFQ sample at 10 ms exhibits EPR spectra with *g* = 2.008. Unlike the case of the nucleotide-deficient wt Arf1, wt Arf1 possesses the nucleotide in its nucleotide binding site. Because the *g* = 2.008 EPR spectrum reflects the presence of the lone Cys-S^•^ (see above), the source of the *g* = 2.008 EPR signals is likely to be the lone Cys-S^•^. However, another EPR signal of the wt Arf1 RFQ sample with *g* = 2.004 appeared at 20 ms and above (Fig. 6*E*). The *g* = 2.004 EPR spectrum was isolated by the deconvolution of the EPR spectra at 20, 50, and 80 ms from the EPR spectra at 10 ms of the wt Arf1 RFQ sample (Fig. 6*E*). According to the radical action-based mechanism, the bound G^•+^ and free G^•^ nucleotides, in addition to the lone Cys-S^•^, were predicted to form during the wt Arf1 redox response. If so, given that the *g* = 2.008 EPR spectrum was assigned to the lone Cys-S^•^ (see above), the deconvoluted EPR spectra with *g* = 2.004 can be assigned to the bound G^•+^ and free G^•^ nucleotides. This *g* value assignment is also comparable to the *g* values of the bound G^•+^ and free G^•^ nucleotides in other systems (65, 66). However, the *g* values of the G^•+^ and G^•^ nucleotides in other systems such as DNA were very similar (66). Likewise, the *g* values of the deconvoluted spectra of the wt Arf1 RFQ sample at different times were almost indistinguishable. Thus, we could not specify the fractional contribution of the bound G^•+^ and free G^•^ nucleotides in the *g* = 2.004 EPR spectra. Notwithstanding, the *g* = 2.004 EPR spectrum was not observed in the deconvoluted spectra at 20 ms of the nucleotide-deficient wt Arf1 RFQ sample from the spectra at 10 ms (Fig. 6*F*). The nucleotide-deficient wt Arf1 lacks the bound guanine nucleotide, which is the precursor of the bound G^•+^ and free G^•^ nucleotides. Therefore, the absence of the *g* = 2.004 EPR spectra in the case of the nucleotide-deficient wt Arf1 RFQ EPR sample (Fig. 6*F*) supports the assignment of the deconvoluted *g* = 2.004 EPR spectra to the bound G^•+^ and free G^•^ nucleotides.

As analyzed above, although the nucleotide-deficient wt Arf1 lacks a nucleotide in its nucleotide binding site, it still exhibited the *g* = 2.008 lone Cys-S^•^ EPR spectra but not the *g* = 2.004 EPR spectra at 10 ms and 20 ms (Fig. 6*A*). These results suggest that the lone Cys-S^•^ can be produced without the presence of the precursor nucleotide for the production of the bound G^•+^ and free G^•^ nucleotides under the experimental conditions. In contrast, the C159S Arf1 sample at 10 ms and 20 ms lacked any EPR signal (Fig. 6*F*). Although C159S Arf1 lacks the lone Cys, it still possesses a guanine nucleotide in its nucleotide binding site. The lack of an EPR signal from the C159S Arf1 sample thus suggests that, without the presence of the lone Cys that is the precursor of the lone Cys-S^•^, the nucleotide in C159S Arf1 cannot be oxidized by a redox agent such as O_2_^•−^ or ^•^NO_2_ to produce the bound G^•+^ and G^•^ nucleotides. Accordingly, the lone Cys-S^•^ production in the Arf1 redox response is a prerequisite for the production of the bound G^•+^ and free G^•^ nucleotides, while the opposite is not true. Therefore, in the Arf1 redox response, the lone Cys-S^•^ is likely formed first, followed by the bound G^•+^ and free G^•^ nucleotides.

Figure 6*E* shows that the intensity of the deconvoluted *g* = 2.004 EPR spectra of the bound G^•+^ and free G^•^ nucleotides increased over time, but that of the *g* = 2.008 lone Cys-S^•^ EPR spectra decreased. The intensity of the EPR signal reflects the quantity of EPR species such as radical components. These results thus suggest that the amount of the lone Cys-S^•^ component first increased and then decreased in the RFQ wt Arf1 EPR samples. This behavior was accompanied by an increase in the content of the bound G^•+^ and free G^•^ nucleotide components. These results suggest that during the wt Arf1 redox response, there was a radical transit from the lone Cys-S^•^ to the bound G^•+^ and free G^•^ nucleotides over time. This interpretation is consistent with the notion that in the wt Arf1 redox response, the lone Cys-S^•^ is produced first, followed by the bound G^•+^ and free G^•^ nucleotides. However, the increase in the bound G^•+^ and free G^•^ nucleotides is not proportional to the decrease in the lone Cys-S^•^. The most plausible explanation for this disproportionate radical transit is that, during the Arf1 redox response, the lone Cys-S^•^ was continuously replenished by the reaction of the lone Cys with the redox agent while it was depleted by the oxidation of the bound guanine nucleotide to produce the bound G^•+^ and free G^•^ nucleotides. If so, the quantity of the lone Cys-S^•^ in the Arf1 redox response was more likely to be in a steady state when the bound G^•+^ and free G^•^ nucleotides were produced.

### Redox-dependent Arf1 functions in cells

Wt HO-8910 cells overexpress Arf1, which controls cell motility (67). Wt HO-8910 cells are shown to express NOX1 and NOX4, which produce a redox agent O_2_^•−^ (68, 69). With this, in conjunction with the redox-sensitive features of wt Arf1, a potential novel redox-dependent regulation pathway for the motility of wt HO-8910 cells can be postulated: O_2_^•−^ produced from NOX1 and NOX4 induces the Arf1 redox response to produce active Arf1, which in turn promotes the migration of wt HO-8910 cells. In this case, the potential redox-dependency of the motility of wt HO-8910 cells can be a reporter of the redox-dependent function of Arf1. The potential cell motility regulation by the Arf1 redox response associated with the lone Cys was thus examined using HO-8910 cells. This examination is critical to evaluate whether the redox-sensitive features of Arf1 apply to cell functions.

**Figure 7:**
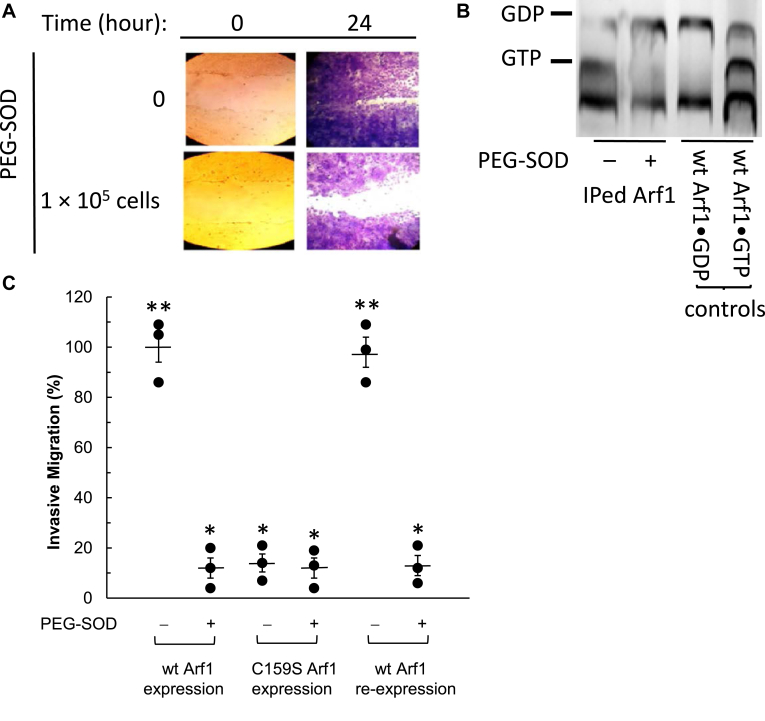
**Determination of the redox-dependent HO-8910 cell migration associated with Arf1 activity.** The redox-dependent cell motilities of wt cells assessed by a wound scratch assay (*A*) associated with the wt Arf1 activity determined by TLC analysis (*B*) are shown. The comparative redox-dependent invasive migrations of wt, C159S-expressed, and wt Arf1 reexpressed cells (*C*) are also shown. The change in the nucleotide-binding status of wt Arf1 in wt cells treated with and without PEG-SOD was studied *via* a wound scratch assay for the cell migration and TLC analysis as described in the experimental procedures section. The invasive cell migration assay for wt, C159S-expressed, and wt Arf1 reexpressed cells in the presence and absence of PEG-SOD was also measured as described in the experimental procedures section. The mean value of the triplicate invasive cell-migration count of wt HO-8910 cells in the absence of PEG-SOD was set to 100%, and other cell migration counts were normalized against the invasive cell migration count of wt HO-8910 cells in the absence of PEG-SOD. The invasive cell migration data in this figure represent the mean values of measurements made in triplicate, and each vertical error bar reflects the standard error. The Tukey ANOVA test results were as follows: ∗, *p* < 0.01, *versus* the invasive cell migration count of wt HO-8910 cells in the absence of PEG-SOD; and ∗∗, *p* < 0.01, *versus* the invasive cell migration count of wt HO-8910 cells in the presence of PEG-SOD. Arf, ADP-ribosylation factor; PEG-SOD, polyethylene glycol-superoxide dismutase.

Figure 7*A* shows that the wt HO-8910 cells treated with polyethylene glycol-superoxide dismutase (PEG-SOD) had a slower cell migration rate than the untreated wt HO-8910 cells. Figure 7*B* further shows that the active GTP-bound wt Arf1 was less populated in the PEG-SOD-treated wt HO-8910 cells than in the untreated control wt HO-8910 cells. PEG-SOD eliminates cellular O_2_^•−^. Given that wt Arf1 is redox-sensitive and that its active form is essential for the motility of wt HO-8910 cells, the results can be interpreted as follows: The removal of the cellular O_2_^•−^ diminishes the redox response of wt Arf1, which in turn limits the production of active wt Arf1 and, consequently, limits the migration of wt HO-8910 cells. There are, however, other possibilities that the removal of the cellular O_2_^•−^ may indirectly affect the function of regulatory proteins such as GEFs and GAPs. O_2_^•−^ may stimulate the GEF action or inhibit the GAP action. If then, when the cellular O_2_^•−^ is removed, it reverses the stimulatory GEF or inhibitory GAP activity, populating inactive wt Arf1 in cells.

Figure 7*C* shows that, although the cellular O_2_^•−^ was not eliminated because of the lack of treatment with PEG-SOD, the cell migration rate of the C159S Arf1-expressed HO-8910 cells was slower than that of wt HO-8910 cells. Also, in contrast to the wt HO-8910 cells, the C159S Arf1-expressed HO-8910 cells did not further decrease their migration when treated with PEG-SOD (Fig. 7*C*). Considering that the lone Cys is responsible for the wt Arf1 redox response, these results can be accounted for in the following way: regardless of the availability of O_2_^•−^, lacking the lone Cys in C159S Arf1 disables the Arf1 redox response to produce active Arf1 and consequently restricts cell motility. The C159S Arf1-expressed HO-8910 cells contain only one Cys mutation on Arf1, but no mutations on other Arf1 regulatory proteins such as GEFs and GAPs. Thus, it was unlikely that the observed impairment of the Arf1 redox response by C159S Arf1 expression and the subsequent restriction of cell migration were caused by changes in other potential indirect redox effects, such as those mediated by or linked to binding interactions with Arf1 regulatory proteins. These notions suggest that replacing C159S Arf1 with the lone Cys-containing wt Arf1 may allow it to restore the Arf1 redox response and populate active wt Arf1, which then permits wt HO-8910 cells to migrate. Figure 7*C* consistently shows that the cell migration rate of wt Arf1 reexpressed HO-8910 cells was comparable to that of wt HO-8910 cells. The PEG-SOD treatment yet again could then limit the cell migration rate of the wt Arf1 reexpressed HO-8910 cells, as seen in wt HO-8910 cells treated with PEG-SOD (Fig. 7*C*). Taken together, these findings and analyses impart a novel example of the biological relevance of the Arf1 redox response with its lone Cys, in which the lone Cys-dependent Arf1 redox response in cellular processes promotes motility in wt HO-8910 cells.

## Discussion

This study first reports the previously unknown redox dependency of the Arf1 function. The redox agent ^•^NO_2_, which is a reaction product of ^•^NO with O_2_, as well as O_2_^•−^ could elicit the redox response of Arf1 to produce active Arf1. The activated Arf1 was also shown to be competent for enhancing Arf1-dependent cell functions such as cell migration.

### Novel Arf-specific redox motif

This study shows that the lone Cys, which is the only Cys residue of Arf1, is redox sensitive. This unique lone Cys, followed by Ala and Thr residues, which we termed the CAT motif, is sequentially conserved in many Arf family GTPases, including Arf2, 3, 4, 5, and 6. Furthermore, the lone Cys in the Arf CAT motif is novel as its sequence does not align with any sequences of the reactive Cys in the Ras NKCD and Rho GX_4_GK(S/T)C/ECS motifs. All of the CAT motif-containing Arf GTPases inherently lack the critical perpendicular redox-conduit Phe. Thus, this redox motif configuration is the simplest yet discovered, comprising only one Cys without the perpendicular redox-conduit Phe.

### Proposed mechanism for the Arf1 redox response

**Figure 8:**
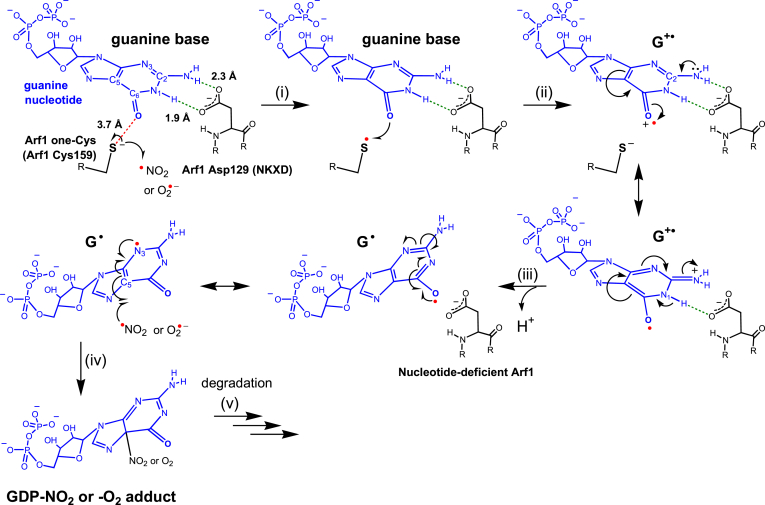
**Proposed mechanism for the redox action on Arf1 nucleotide dissociation**. Molecular steps are shown for the ^•^NO_2_- or O_2_^•−^-mediated sequential formation of the wt Arf1 lone Cys-S^•^ and bound G^•+^ and free G^•^ nucleotides followed by quenching of the free G^•^ nucleotide with another redox agent, ^•^NO_2_ or O_2_^•−^, to produce the GDP-NO_2_ or GDP-O_2_ adduct. The GDP-O_2_ and GDP-NO_2_ adducts have different degradation products. GDP-NO_2_ degrades to 5-guanidino-4-nitroimidazole diphosphate (NIm-DP) (47). The GDP-O_2_ adduct degrades mainly to DIz-DP (first reported in this study) but also to AIm-DP and IIm-DP (46). Arf, ADP-ribosylation factor.

This study shows that the Arf1 redox response was coupled with a release of the nucleotide adduct, but not the unmodified nucleotide. The presence of the transient radical lone Cys-S^•^ followed by the bound G^•+^ and free G^•^ nucleotides was also detected during the Arf1 redox response. However, modifications of reactive lone Cys thiols were not detected in the Arf1 redox response regardless of the presence or absence of a one-electron acceptor such as NAD^+^ and transition metals. Given these results, we propose a radical action-based Arf1 redox response while dismissing the role of the lone Cys-thiol modification in the Arf1 redox response: (Fig. 8): (i) a redox agent, such as ^•^NO_2_ or O_2_^•−^, oxidizes the lone Cys to produce the lone Cys-S^•^; (ii) the lone Cys-S^•^ then oxidizes directly the bound nucleotide to produce the bound G^•+^ nucleotide, and (iii) the bound G^•+^ nucleotide is then deprotonated and released from Arf1 as a free G^•^ nucleotide, resulting in the production of the nucleotide-deficient Arf1; (iv) another redox agent such as ^•^NO_2_ or O_2_^•−^ quenches the free G^•^ nucleotide to produce a nucleotide adduct such as the GDP-NO_2_ or -O_2_ adduct; and (v) the nucleotide adduct is then further converted into its degradation forms.

In cells, whenever the Arf1 redox response produces the nucleotide-deficient Arf1, the cellularly abundant free GTP binds to the nucleotide-deficient Arf1 to produce active GTP-bound Arf1.

### Putative mechanistic aspects underlying the Arf1 redox response

Chemical properties, including the redox potentials of the redox agents ^•^NO_2_ and O_2_^•−^ as well as the free Cys and free guanine nucleotide, are known. The Arf1 crystal structure is also reported. In combination, the fundamentals of the redox reaction and coupling events between the lone Cys and bound nucleotide, which precede the free nucleotide adduct formation in the model mechanism (Fig. 8), can be postulated as follows.

#### Step (i), lone Cys-S^•^ formation

The redox potentials of ^•^NO_2_/NO_2_^–^ (∼1.0 V *versus* NHE) and O_2_^•−^/H_2_O_2_ (∼0.9 V *versus* NHE) are slightly higher or similar to that of free Cys-S^•^/free Cys thiolate (Cys-S^–^) (∼0.9 V *versus* NHE) (70, 71, 72, 73). The sulfur atom of the lone Cys side chain in the Arf1 crystal structure is solvent exposed (Fig. 1). Thus, the redox potentials of the solvent-exposed lone Cys-S^•^/lone Cys-S^–^ and free Cys-S^•^/Cys-S^–^ are unlikely to differ. The analysis suggests that the lone Cys-S^–^ can serve as a reductant for ^•^NO_2_ and O_2_^•−^ so that ^•^NO_2_ or O_2_^•−^ can oxidize the lone Cys-S^–^ to produce the lone Cys-S^•^ as well as NO_2_^–^ or H_2_O_2_.

#### Step (ii), bound G^•+^ nucleotide formation

The Arf1 crystal structure also shows that the C_6_O site of the bound guanine nucleotide faces the sulfur atom of the Arf1 lone Cys side chain at a distance of 3.7 Å (Fig. 1). Because of their proximity, the bound guanine nucleotide might undergo redox coupling with the lone Cys through its C_6_O site. The redox potential of free G^•+^/guanine base (∼1.3 V versus NHE) is known (74) and is higher than the putative redox potential of the solvent-exposed lone Cys-S^•^/lone Cys-S^–^ (∼0.9 V versus NHE) (see above). However, to proceed with the lone Cys-mediated oxidation of the bound guanine nucleotide to produce the bound G^•+^ nucleotide during the Arf1 redox response, the redox potential of the bound G^•+^/guanine nucleotide must be lower than that of the lone Cys-S^•^/Cys-S^–^ of Arf1. Thus, the free guanine nucleotide might have a redox potential lowering mechanism when it binds to Arf1 to form the bound guanine nucleotide. The Arf1 crystal structure shows that, like other small GTPases, a proton on C_2_-N (C_2_-NH_2_) and the proton on N_1_ (N_1_-H) of the bound guanine nucleotide are, respectively, 2.3 and 1.9 Å apart from the side chain O atoms of Asp129 (Arf1 numbering) in the NKXD motif of Arf1 (Fig. 8). Such close atomic distances suggest that the bound guanine nucleotide has strong hydrogen-bonding interactions with the side chain of Asp129. These interactions then implement additional resonance on the bound guanine nucleotide that would establish the redox potential of the bound G^•+^/guanine nucleotide below that of the lone Cys-S^•^/Cys-S^–^ of Arf1. The lowered redox potential of the bound G^•+^/guanine nucleotide permits the formed lone Cys-S^•^ to oxidize the bound guanine nucleotide through its C_6_O site, resulting in the production of the bound G^•+^ nucleotide.

#### Step (iii), free G^•^ nucleotide formation

The bound G^•+^ nucleotide is tautomerized, disrupting its hydrogen-bonding interactions with the Arf1 Asp129 side chain. It is subsequently liberated from Arf1 as a free G^•^ nucleotide. Its release produces the nucleotide-deficient Arf1.

#### Step (iv), nucleotide adduct formation

Upon its release from Arf1, the C_5_ site of the free G^•^ nucleotide becomes exposed to solvent, which in turn permits the free G^•^ nucleotide to be quenched by another redox agent such as ^•^NO_2_ or O_2_^•−^ to produce the guanine nucleotide adduct. This quenching reaction depletes the free G^•^ nucleotide, which may thermodynamically help facilitate steps (ii) and (iii), resulting in the promotion of the guanine nucleotide dissociation from Arf1 as a form of the guanine nucleotide adduct.

#### Step (v), nucleotide adduct degradation

Because it is unstable, the guanine nucleotide adduct is expected to further degrade to its stable forms. The GDP-NO_2_ adduct degraded to NIm-DP (47), whereas the GDP-O_2_ adduct degraded to DIz-DP (this study), as well as AIm-DP and IIm-DP (46).

### Implications of the redox response mechanism of Arf1 for those of Ras and Rho GTPases

Although the redox motif configuration of Ras and Rho GTPases differs from that of Arf1 (Fig. 1), the same radical intermediates, the reactive Cys-S^•^ and bound G^•+^ and free G^•^ nucleotides, were also proposed to be involved in the redox events for the Ras and Rho redox responses (5, 47). However, the presence of these radical intermediates during their redox responses has not been validated. Thus, the RFQ EPR-based identification of the transient lone Cys-S^•^ and bound G^•+^ and free G^•^ nucleotides in the Arf1 redox response opens the possibility that these radical equivalents also occur in the Ras and Rho redox responses. Further studies that probe the potential radical intermediates in the Ras and Rho redox responses are necessary to comprehensively understand the radical action-based redox response mechanisms of Ras and Rho proteins.

It was proposed that the unique spatial position of the perpendicular Phe in Ras and Rho (Fig. 1, *A* and *B*) enables it to serve as a redox conduit between the reactive Cys side chain and the bound guanine nucleotide (5, 46, 47). However, the proposed function of the perpendicular Phe is problematic because the redox potential of one electron-oxidized Phe (Phe^•+^)/Phe (∼2.0 V *versus* NHE) (75, 76) is considerably higher than that of the reactive Cys-S^•^/Cys-S^−^ (presumably ∼0.9 V *versus* NHE, see above) and the bound G^•+^/guanine nucleotide (presumably < ∼0.9 V *versus* NHE, see above). Consequently, the redox-conduit function of the perpendicular Phe encounters an uphill step that is thermodynamically unfavorable (see details in the Supporting Information section). Yet, there are some reports for the occurrence of the thermodynamically unfavorable uphill-electron transfer in some biological systems (77, 78). An attempt to explain the uphill process associated with the proposed electron conduit function of the perpendicular Phe in the Ras and Rho redox responses used Marcus theory (5).

In the proposed Arf1 redox response mechanism, the reactive lone Cys-S^•^ directly interacts with the bound guanine nucleotide without the redox-conduit function of the perpendicular Phe to produce the lone Cys-S^−^ and the bound G^•+^ nucleotide. Intriguingly, like Arf1, the reactive Cys sidechain of Ras faces C_6_O of the bound guanine nucleotide at a distance of ∼6.7 Å (Fig. 1*A*), which was ignored previously. In the case of Rho GTPase, a newly found reactive Cys in the ECS module of the GX_4_GK(S/T)C/ECS motif faces the C_6_O site of the bound guanine nucleotide at a distance of ∼6.6 Å (Fig. 1*B*). When combined, it is possible that, as with the case of Arf1, the formed reactive Cys-S^•^ in Ras and Rho GTPases directly, but not through the perpendicular redox-conduit Phe, oxidizes the bound guanine nucleotide to produce the reactive Cys-S^−^ and the bound G^•+^ nucleotide. This direct oxidation path does not necessarily involve the thermodynamically unfavorable uphill redox-conduit path associated with the perpendicular Phe in the Ras and Rho GTPases.

Although the direct oxidation path of the Ras and Rho redox responses appears plausible, the reason that F28L Ras lacks the Ras redox response remains unclear (47). One of the previously overlooked potential reasons is that the displaced Leu side chain in F28L Ras weakens the hydrogen-bonding interactions of the bound guanine nucleotide with the side chain of Ras Asp129 (see details in the Supporting Information section). In essence, F28L Ras showed substantially weak binding interactions with the guanine nucleotide (79). This fails to reduce the redox potential of the bound G^•+^/guanine nucleotide below that of the reactive Cys-S^•^/Cys-S^−^ (presumably ∼0.9 V *versus* NHE, see above). As a result, the formed reactive Cys-S^•^ is unable to oxidize the bound guanine nucleotide to produce the G^•+^ nucleotide, resulting in no redox response from F28L Ras. These analyses suggest an alternative possibility that the absence of the F28L Ras redox response can be due to the failure of the displaced Leu to lower the redox potential of the bound guanine nucleotide, rather than the absence of the redox-conduit path associated with the perpendicular Phe.

Still, however, because the reactive Cys in Ras and Rho faces the perpendicular Phe side chain, the perpendicular Phe has a potential role in Ras and Rho redox response. One of the possibilities is the potential redox-mediated perturbation of the interface between the sidechains of the perpendicular Phe and the reactive Cys, which would have an effect on the guanine nucleotide binding interaction with Ras and Rho (see details in the Supporting Information section). Thus, such a redox-dependent side chain interface perturbation is necessary to study for a better understanding of any potential role of the perpendicular Phe in the redox response of Ras and Rho GTPases.

## Experimental procedures

All chemicals used in the kinetic studies, including GDP and GTP and their fluorescent-tagged forms such as 2'-(or-3′)-O-(N-methylanthraniloyl) guanosine 5′-diphosphate (mtGDP) and 2'-(or-3′)-O-(N-methylanthraniloyl) guanosine 5′-triphosphate (mtGTP), were of the finest grade.

### Transition metal-free experimental conditions

Transition metal ions (*e*.*g*., Fe^2+^/Fe^3+^) are often involved in the conversion of one redox agent to another (80, 81). To exclude this conversion, transition metal-free buffers were prepared as described below and then used for kinetic, mass spectroscopy, and EPR studies unless otherwise noted.

To minimize the transition metal contamination from vials that store buffer and protein samples, these vials were immersed in 1 N HCl overnight and then rinsed with distilled water before use. A 100 mM phosphate buffer (pH 7.4) was filtered through a Bio-Rad Chelex-100 cation exchange resin to eliminate any traces of transition metals. The highest purity grade of NaCl and EDTA was then added to the metal-chelated phosphate buffer to produce a transition metal-free sample buffer that contains 200 mM NaCl and 1 mM EDTA in 100 mM phosphate buffer (pH 7.4). The highest purity grade of MgCl_2_ and GDP was further added to the sample buffer to produce a transition metal-free assay buffer that comprises 100 μM GDP, 2 mM MgCl_2_, 200 mM NaCl, and 1 mM EDTA in 100 mM phosphate buffer (pH 7.4).

### Anaerobic experimental conditions

Some redox agents react with O_2_ to produce their oxide forms (5, 82). To avoid the reactions of the redox agents with ambient O_2_, anaerobic buffer treated with a specific quantity of a redox agent and anaerobic Arf1 protein samples were prepared as described below and then used for the kinetic, mass spectroscopy, and EPR analyses under anaerobic conditions, unless specified otherwise.

Assay solutions with various concentrations of a redox agent were prepared in a N_2_-filled anaerobic glovebox (O_2_ < 3 PPM). For this preparation, ^•^NO (98.5%, Sigma-Aldrich) and ^•^NO_2_ (≥99.5%, Sigma-Aldrich) gas cylinders were placed in an anaerobic glovebox. Vials containing the assay buffer as well as powder potassium superoxide (source of O_2_^•−^, 100%, Sigma-Aldrich), stock solution H_2_O_2_ (30% w/w in H_2_O, Sigma-Aldrich), and stock solution peroxynitrite (160–200 mM in 4.7% NaOH, Sigma-Aldrich) were vacuumed, filled with N_2_ gas, and then placed in the anaerobic glovebox. To prepare an assay solution with a specific concentration of ^•^NO or ^•^NO_2_ and then conserve its content until use, a syringe was used to deliver ^•^NO or ^•^NO_2_ gas into a serum stopper-sealed assay cuvette containing assay buffer (1 ml). Notably, unlike others, peroxynitrite was prepared in 4.7% NaOH. However, as with others, only a small amount of the peroxynitrite stock solution (*e*.*g*., 10 μl) was transferred to the assay buffer (1 ml) containing the 100 mM phosphate buffer (pH 7.4). As a result, the assay solution containing peroxynitrite maintained a pH of 7.4. An aliquot of the assay solutions with ^•^NO or ^•^NO_2_ was withdrawn from the assay cuvettes using a syringe, and its ^•^NO or ^•^NO_2_ content was measured using the hemoglobin-coupled assay (33, 83). The same was applied to prepare O_2_^•−^-, H_2_O_2_-, peroxynitrite-containing assay solutions in serum stopper-sealed assay cuvettes, except a fraction of the O_2_^•−^, H_2_O_2_, or peroxynitrite stock solution instead of ^•^NO and ^•^NO_2_ gas is transferred using a syringe. An aliquot of assay solutions with O_2_^•−^, or H_2_O_2_, or peroxynitrite was also withdrawn using a syringe, and then its O_2_^•−^, H_2_O_2_, or peroxynitrite content was quantified using the luminol-based O_2_^•−^ assay kit (Sigma-Aldrich), the chromogenic Fe^2+^ oxidation-based H_2_O_2_ assay (Sigma-Aldrich), or the hemoglobin-coupled assay (33, 83), respectively. Our assay system limits many factors, such as transition metals (and O_2_ when necessary), that facilitate the conversion of these redox agents into other forms. However, still some of these redox agents can be converted into other forms in a short time. In particular, among these redox agents, O_2_^•−^ rapidly disproportionates into O_2_ and H_2_O_2_ (84, 85). To determine the redox agent concentration measurement and reaction onset time were synchronized. For example, the time spent withdrawing an aliquot of the assay solutions with O_2_^•−^ using a syringe and then injecting it into the luminol-containing assay kit was 3 s. The assay was likewise initiated at exactly 3 s following the aliquot withdrawal for the measurement of O_2_^•−^ content.

### Anaerobic kinetic, mass spectroscopy, and EPR Arf1 protein preparations

Full-length wt Arf1 and its mutants, C159S, Y/F, and Y/F C159S Arf1, were expressed in and purified from *Escherichia* *coli*. These Arf1 mutants were produced by using a site-directed mutagenesis kit (Thermo Fisher Scientific). The as-purified Arf1 proteins were mostly in the GDP-bound form (>99% GDP-bound Arf1). For further uses, the as-purified Arf1 proteins were processed to mtGDP-bound form, nucleotide-deficient form, and simply buffer exchanged form. To produce the GDP-bound Arf1 in a sample buffer, as-purified Arf1 proteins were passed without further treatment through a G-25 column that was equilibrated with the sample buffer. To produce mtGDP-bound Arf1, as-purified Arf1 (∼1 μM) was treated with 400 mM (NH_4_)_2_SO_4_ for 10 min. Excess mtGDP (1 mM) was then added, followed by quenching with 10 mM MgCl_2_. The mtGDP-quenched Arf1 was then run through a G-25 column equilibrated with the sample buffer to collect the mtGDP-bound Arf1 in the sample buffer. To produce nucleotide-deficient Arf1, as-purified Arf1 was treated with 400 mM (NH_4_)_2_SO_4_ for 10 min. The treated Arf1 was then applied onto a G-25 column that was equilibrated with 50 mM (NH_4_)_2_SO_4_ in the sample buffer without GDP. The nucleotide-deficient Arf1 in the sample buffer with 50 mM (NH_4_)_2_SO_4_ but without GDP was collected. Nucleotide-deficient Arf1 in this condition was stable up to its concentration of <100 μM. All of these Arf1 proteins were further concentrated using Amicon Centricon (10 kDa cutoff). These concentrated Arf1 samples were then placed in serum stopper-sealed vials. These vials were then vacuumed and replenished with N_2_ three times before the Arf1 protein samples were used in experiments under O_2_-free anaerobic conditions.

### Kinetic analysis

Dissociation of mtGDP from Arf1 decreases mant fluorescent intensity. Accordingly, to measure whether a redox agent enables GDP dissociation from Arf1 under anaerobic conditions, anaerobically prepared mtGDP-loaded Arf1 was transferred to a serum stopper-sealed assay cuvette that contains anaerobic assay solution with a specific redox agent. Airtight syringes flushed with N_2_ were used for this transfer to avoid any O_2_ contamination. The changes in mant fluorescent intensity at 460 nm were then monitored over time using a PerkinElmer LS55 fluorometer. The same experiments were also performed under the aerobic conditions. For these experiments, atmospheric O_2_ was injected using a syringe before transferring mtGDP-loaded Arf1 to serum stopper-sealed assay cuvettes containing anaerobic assay buffer with a redox agent. The following mant fluorescent intensity changes were also monitored over time using the fluorometer. In contrast to the mtGDP dissociation from Arf1, association of mtGDP to Arf1 increases mant fluorescent intensity.

To measure whether a redox agent enables GDP association to Arf1 under anaerobic conditions, anaerobically prepared nucleotide-deficient Arf1 and free mtGDP were transferred to a serum stopper-sealed assay cuvette that contains the anaerobic assay solution with a specific redox agent. Because free mtGDP was introduced separately, the assay solution for this analysis lacked GDP. As with the Arf1 mtGDP dissociation assay, an airtight syringe was used for all sample transfers. To generate aerobic conditions, atmospheric O_2_ was added before the transfer of the protein samples to the anaerobically sealed assay cuvettes. The change in mant fluorescent intensity over time was also measured for the Arf1 mtGDP dissociation assay.

### Mass spectrometric analysis

For all mass spec analyses, GDP-bound Arf1 samples were used instead of mtGDP-bound Arf1 samples. Sample buffer that lacks GDP was also used instead of assay buffer with GDP to avoid confusion in the detection of the potential unmodified GDP dissociation from Arf1 with the preexisting GDP in the assay system. However, for comparison, the mass spectroscopy sample preparation conditions were identical to the kinetic analysis conditions. Accordingly, GDP-bound Arf1 was treated with a redox agent for 2 min in a serum stopper-sealed assay cuvette containing the anaerobic sample buffer. When necessary, as in the kinetic analysis, atmospheric O_2_ was added to produce aerobic experimental conditions. Before exposing the resultant redox reaction products in a sealed assay cuvette to air, they were purged with N_2_ gas for 5 min. The sample buffer containing the Arf1 redox reaction products was then separated from the Arf1 protein using Amicon Centricon (10 kDa cutoff).

To analyze the nucleotide released, the sample buffer fraction of the Arf1 redox reaction was mixed with formic acid (0.1%) and methanol (50%) without any further treatment. To analyze the Arf1 thiol modification, the Arf1 protein fraction of the Arf1 redox reaction was washed with fresh sample buffer three times and then digested with trypsin. As a control, the fresh Arf1 sample in the sample buffer was also treated with iodoacetate (10 mM) before the trypsin digestion. These trypsin-digested samples were further mixed with formic acid (0.1%) and methanol (50%). The sample buffer and Arf1 fractions were then analyzed with ESI-MS in positive ion mode ([Mass + H]^+^) on a Shimadzu LCMS-9030 mass analyzer. Because the ESI-MS samples were acidified with formic acid, which produces single-charged ions, the resulting molecular weights determined by ESI-MS were 1 Da greater than the molecular weights for the same molecules at neutral pH.

### Rapid-mix freeze quench EPR analysis

All RFQ Arf1 EPR samples were prepared using a KinTek RFQ-3 apparatus. All syringes attached to the end lines of the apparatus were airtight and were purged with N_2_ gas before use. Anaerobically prepared nucleotide-deficient or GDP-bound Arf1 (100 μM) was rapidly mixed with O_2_^•−^ (100 μM) in the assay buffer at room temperature, and the mixing ratio was 1:1 (v/v). The reaction mixtures (∼400 μl) were quenched at various times (10–80 ms) by spraying them into liquid ethane at −170 °C. The resulting precipitate was collected and then packed into the quartz EPR tube bottoms. The Arf1 RFQ samples were used for X-band EPR analyses using Bruker EMXplus.

### Cell culture and migration analysis

Cells die without the transition metals and under anaerobic conditions. Thus, the transition metal-free and anaerobic experimental conditions were not applied to the cell-based analysis. HO-8910 cells (Cells Online) were validated by single tandem repeat analysis before use. There was no contamination of other cell lines. The cells were not contaminated with other cell lines and were clear of *mycoplasma*. The cells were cultured in RPMI 1640 media supplemented with fetal bovine serum (10%) and penicillin (100 units/ml) in a humidified CO_2_ (5%) incubator at 37 °C. To produce wt Arf1 knockdown and then express C159S Arf1 or reexpress wt Arf1 in cells, wt Arf1-knockdown cells were first generated with lentivirus-mediated short-hairpin RNAs that target the 3′ untranslated regions of the endogenous wt Arf1 sequence. This knockdown was validated by using quantitative real-time PCRs and Western blot analysis. The wt Arf1 knockdown 60 cells were then stably transfected with the full-length C159S or wt Arf1 construct in the pMSCV vector per the specifications of the manufacturer (Thermo Fisher Scientific).

#### Wound scratch migration assay

Cells were grown on a 24-well plate at 37 °C for 24 h. Once cells formed a confluent monolayer on the plate, a "line" or "scratch" was drawn on the underside of each well before the wound scratch migration assay (86, 87). This line served as a fiducial marker for analyzing the wound areas. The nonadhering cells were washed off with phosphate-buffered saline and then treated with and without PEG-SOD (10,000 units). Cells on the plate were visualized after 24 h by staining with tris(4-(dimethylamino)phenyl)methylium chloride. The activity of wt Arf1 in wound scratch migration assay cell samples was analyzed with TLC as described previously (88), except that a monoclonal anti-Arf1 antibody (Invitrogen) was used for the wt Arf1 immunoprecipitation instead of the Ras antibody. As controls, GTP- and GDP-bound purified wt Arf1 were used for this TLC analysis.

#### Cell invasion assay

The invasive migration of HO-8910 cells was measured using the colorimetric-based CHEMICON cell invasion assay kit (Millipore Sigma). Cells were suspended in a serum-free medium at a concentration of ∼5 × 10^5^ cells/ml. The cell suspensions (0.2 ml) were added to the top chamber, whereas the cell-free media (0.5 ml) with fetal bovine serum (10%) were placed in the lower chamber. The cells in the top chamber were treated with a serum-free medium (0.1 ml) with or without PEG-SOD (10,000 units) and then incubated for 24 h at 37 °C in an incubator with CO_2_ (5%) at 37 °C. The cell migration from the upper chamber to the lower chamber was then quantified colorimetrically following the manufacturer's protocols.

## Data availability

All data are contained within the manuscript.

## Supporting information

This article contains supporting information.

## Conflict of interest

The authors declare that they have no conflicts of interest with the contents of this article.
