# Supplementary material for: Redox response feature and mechanism of Arf1 and their implications for those of Ras and Rho GTPases
Source: J Biol Chem. 2025 May 21;301(6):110269. doi: 10.1016/j.jbc.2025.110269 (PMC12192700; doi:10.1016/j.jbc.2025.110269)
Supplement: Supporting Information [file mmc1.docx]

**Supporting Information**

**Fig. S1**. *Proposed radical action-based mechanism for the Ras redox response*. The Ras-bound guanine nucleotide and its hydrogen bonding interactions with Asp119 are depicted. The perpendicularly positioned putative redox-conduit Phe over the bound guanine base is also shown. The "wave" symbol represents the vertical binding interactions of the bound guanine nucleotide base with the perpendicular Phe of Ras. The mechanistic steps of the radical action-based nucleotide dissociation from Ras with its NKCD motif (Fig. S1A) are shown (**A**). This mechanism was adapted from the previously proposed mechanism (1) by changing the redox-sensitive Ras Cys-SH to its thiolate form Ras Cys-S^−^, which reacts with ^•^NO_2_ to produce Ras Cys-S^•^ (2). This was because, compared to free Cys-S^•^/free Cys-S^–^ (~0.9 V vs. NHE), free Cys-S^•^/free Cys-SH (~1.3 V vs. NHE) has a higher redox potential than that of ^•^NO_2_/NO_2_^–^ (~1.0 V vs. NHE) and O_2_^•−^/H_2_O_2_ (~0.9 V vs. NHE) (3-6). Thus, the redox-sensitive Ras Cys118 sidechain could be Ras Cys-S^−^ rather than Ras Cys-SH, as depicted in this figure. This mechanism applies to the Rho redox response with its GX4GK(S/T)C/ECS motif, as the Rho Cys-S^−^ could exist vicinal to the Rho perpendicular Phe (Fig. S1B) (7,8). The mechanistic steps of the thiol modification-based nucleotide dissociation from Ras associated with its NKCD motif are also shown (**B**). The putative Ras-SNO production mechanism in the presence of an oxidant (NAD^+^, **B1**) or a transition metal (e.g., Cu^2+^, **B2**) that may cause a local conformational change in the Ras nucleotide binding site is also shown.

*Thermodynamic challenge for the redox-conduit function of the perpendicular Phe in Ras and Rho redox response*. The previously proposed redox events between the formed redox-sensitive Cys-S^•^ and the bound guanine base through the perpendicular redox-conduit Phe might occur simultaneously. However, for convenience, they can be divided into two steps (Fig. S2). In Step 1, the redox-sensitive Cys-S^•^ withdraws an electron from the perpendicular Phe to produce the redox-sensitive Cys-S^−^ and one electron-oxidized Phe (Phe^•+^); In Step 2, the formed Phe^•+^ takes an electron from the bound guanine base to produce Phe and the bound G^•+^ nucleotide. The first Phe^•+^ production step (Step 1) is thermodynamically uphill, while the second bound G^•+^ nucleotide production step (Step 2) is downhill (Fig. S2).

**Fig. S2**. *Thermodynamics of the potential Ras and Rho redox events associated with the redox-conduit Phe*. The thermodynamic scheme for the previously proposed Ras and Rho redox events, including their essential redox components and their related redox potentials, is illustrated. The relative redox potentials of the essential redox components involved in the Ras and Rho redox response are as follows: Phe^•+^/Phe (~2.0 V vs. NHE) (9,10) > the redox-sensitive Cys-S^•^/Cys-S^−^ (presumably ~0.9 V vs. NHE, see the main article) > the bound G^•+^/guanine nucleotide (presumably < ~0.9 V vs. NHE, see the main article). The path for the one electron movement from Phe to the redox-sensitive Cys-S^•^ to produce Phe^•+^ and the redox-sensitive Cys-S^−^ (Step 1) and from the bound guanine base to Phe^•+^ to produce Phe and the bound G^•+^ nucleotide (Step 2) are shown with dotted lines.

*Perturbation of the Ras nucleotide-binding interactions in F28L Ras*. In most small GTPases, including Ras, the hydrogen atom at the *para* position of the perpendicular Phe sidechain (Phe sidechain C_4_-H) verges on the π electron-rich bound guanine base (Fig. 1A and 1B). This configuration results in the *n*-π stacking interactions, which have been known for a decade (11-19). The interactions are considered critical for Ras nucleotide binding interactions (20,21).

Beyond the prior attempt to link the *n*-π stacking interactions to the redox events in the Ras NKCD motif (1), no further progress has been achieved in understanding the role of the *n*-π stacking interactions in the redox response of Ras and Rho. In Ras and Rho, the spatial configuration of the perpendicular Phe position over the bound guanine base predicts the feature of orthogonal electron orbital overlaps within the *n*-π stacking interactions, where the electron pair (*n*) of the Phe sidechain C_4_-H partially delocalizes into the antibonding orbital (π*) of the bound guanine base. The partial delocalization is expected to result in orthogonal *n*→π* interactions. The *n*→π* interactions are weak (~0.5 kcal/mol) (22-24). However, they may still be beneficial for keeping the bound guanine base in the best pose to align it with the Ras Asp side chain. This enhances the hydrogen bonding interactions between the bound guanine base and the Ras Asp sidechain. Conversely, the bound guanine base may be released from its pose for the best hydrogen-bonding interactions with the Asp sidechain of F28L Ras if the orthogonal *n*→π* interactions are eliminated by altering the perpendicular Phe with Leu to produce F28L Ras. As a result, the hydrogen-bonding interactions between the Asp sidechain of F28L Ras and the attached guanine nucleotide would be weakened.

Moreover, the Leu sidechain is ~1.0 Å shorter than the perpendicular Phe sidechain. As a result, the short Leu sidechain in the F28L Ras causes the bound guanine base to tilt upward from its normal position associated with the perpendicular Phe. This would widen the hydrogen-bonding interaction distance between the bound guanine nucleotide and the Asp side chain of F28L Ras. This also weakens the hydrogen-bonding interactions between the bound guanine nucleotide and the Asp side chain of F28L Ras. F28L Ras lacks the orthogonal *n*→π* interactions, which is proposed to optimize the hydrogen bonding interactions between the bound guanine base and the Asp sidechain of Ras (see above). When combined, the hydrogen-bonding interactions between the bound guanine base and the Asp side chain in F28L Ras are proposed to be weakened in two ways: the wider hydrogen-bonding interaction distance between the bound guanine base and the Asp side chain and the absence of the orthogonal *n*→π* interactions. This combination would account for the unexpectedly weak binding interaction of the guanine nucleotide with F28L Ras (20,21). The weaker hydrogen-bonding interactions between the binding guanine base and the Asp side chain in F28L Ras may fail to lower the redox potential of the bound guanine base below the redox potential of the redox-sensitive Cys-S^−^. We thus hypothesize that the main cause of the lack of the F28L Ras redox response is not the absence of the redox-conduit path associated with the perpendicular Phe, but rather the failure to lower the redox potential of the bound guanine base below the redox potential of the redox-sensitive Cys-S^−^ in F28L Ras.

It is noteworthy that the hydrogen-bonding interaction distances between the bound guanine nucleotide and the Asp sidechain of Arf1 (1.9 Å and 2.3 Å, PDB 6PTA) are ~32% shorter than those between the bound guanine nucleotide and the Asp sidechain of Ras (2.7 Å and 3.5 Å, PDB 1Q21). Therefore, the strong hydrogen-bonding interactions between the bound guanine nucleotide and the Asp sidechain may be intrinsic to Arf1 even in the absence of the perpendicular Phe. Accordingly, in contrast to Ras and Rho, the absence of the perpendicular Phe in Arf1 might not alter the hydrogen-bonding interactions between the Asp side chain and the bound guanine base. We thus hypothesize that the Arf1 redox response is possible because the bound guanine base in Arf1 has a lower redox potential than the lone Cys-S^−^ without the perpendicular Phe.

*Potential radical-mediated perturbation of the perpendicular Phe position and its effect in the Ras and Rho redox response*. According to the valence-shell electron-pair repulsion (VSEPR) theory, the radical, an unpaired electron, remains an area of electron density, even if its repulsive strength is significantly reduced (25-27). Thus, the generated redox-sensitive Cys-S^•^ may diminish the repulsion between the sidechains of the redox-sensitive Cys and the perpendicular Phe. This decreased repulsion may allow the perpendicular Phe sidechain to swing towards the newly produced redox-sensitive Cys-S^•^. This swing action may disrupt the orthogonal *n*→π* interactions between the Phe sidechain and the guanine-bound nucleotide, leading to guanine dissociation from Ras and Rho. Counting the long and short distances between the perpendicular Phe sidechain and the redox-sensitive Cys of Ras and Rho (Figs. 1A and 1B), respectively, the redox-dependent orthogonal *n*→π* interaction perturbation in Ras is likely minimal, but in Rho it is substantial. The potential role of Phe in Ras and Rho redox responses in this unique aspect has yet to be explored.

**References**

1. Heo, J., Prutzman, K. C., Mocanu, V., and Campbell, S. L. (2005) Mechanism of free radical nitric oxide-mediated Ras guanine nucleotide dissociation. *Journal of molecular biology* **346**, 1423-1440

2. Heo, J. (2011) Redox control of GTPases: from molecular mechanisms to functional significance in health and disease. *Antioxidants & redox signaling* **14**, 689-724

3. Koppenol, W. H., Moreno, J. J., Pryor, W. A., Ischiropoulos, H., and Beckman, J. S. (1992) Peroxynitrite, a cloaked oxidant formed by nitric oxide and superoxide. *Chem Res Toxicol* **5**, 834-842

4. Buettner, G. R. (1993) The pecking order of free radicals and antioxidants: lipid peroxidation, alpha-tocopherol, and ascorbate. *Arch. Biochem. Biophys.* **300**, 535-543

5. Stubbe, J., and van Der Donk, W. A. (1998) Protein Radicals in Enzyme Catalysis. *Chemical Reviews* **98**, 705-762

6. Shafirovich, V., Cadet, J., Gasparutto, D., Dourandin, A., and Geacintov, N. E. (2001) Nitrogen dioxide as an oxidizing agent of 8-oxo-7,8-dihydro-2'-deoxyguanosine but not of 2'-deoxyguanosine. *Chem. Res. Toxicol.* **14**, 233-241

7. Heo, J., Raines, K. W., Mocanu, V., and Campbell, S. L. (2006) Redox regulation of RhoA. *Biochemistry* **45**, 14481-14489

8. Heo, J., and Campbell, S. L. (2005) Mechanism of redox-mediated guanine nucleotide exchange on redox-active Rho GTPases. *The Journal of biological chemistry* **280**, 31003-31010

9. Roy, A., Seidel, R., Kumar, G., and Bradforth, S. E. (2018) Exploring Redox Properties of Aromatic Amino Acids in Water: Contrasting Single Photon vs Resonant Multiphoton Ionization in Aqueous Solutions. *The journal of physical chemistry. B* **122**, 3723-3733

10. Nathanael, J. G., Gamon, L. F., Cordes, M., Rablen, P. R., Bally, T., Fromm, K. M., Giese, B., and Wille, U. (2018) Amide Neighbouring-Group Effects in Peptides: Phenylalanine as Relay Amino Acid in Long-Distance Electron Transfer. *Chembiochem : a European journal of chemical biology* **19**, 922-926

11. Pai, E. F., Kabsch, W., Krengel, U., Holmes, K. C., John, J., and Wittinghofer, A. (1989) Structure of the guanine-nucleotide-binding domain of the Ha-ras oncogene product p21 in the triphosphate conformation. *Nature* **341**, 209-214

12. Brunger, A. T., Milburn, M. V., Tong, L., deVos, A. M., Jancarik, J., Yamaizumi, Z., Nishimura, S., Ohtsuka, E., and Kim, S. H. (1990) Crystal structure of an active form of RAS protein, a complex of a GTP analog and the HRAS p21 catalytic domain. *Proc. Natl. Acad. Sci. U.S.A.* **87**, 4849-4853

13. Kraulis, P. J., Domaille, P. J., Campbell-Burk, S. L., Van Aken, T., and Laue, E. D. (1994) Solution structure and dynamics of ras p21.GDP determined by heteronuclear three- and four-dimensional NMR spectroscopy. *Biochemistry* **33**, 3515-3531

14. Tong, L. A., de Vos, A. M., Milburn, M. V., and Kim, S. H. (1991) Crystal structures at 2.2 A resolution of the catalytic domains of normal ras protein and an oncogenic mutant complexed with GDP. *J. Mol. Biol.* **217**, 503-516

15. Ito, Y., Yamasaki, K., Iwahara, J., Terada, T., Kamiya, A., Shirouzu, M., Muto, Y., Kawai, G., Yokoyama, S., Laue, E. D., Walchli, M., Shibata, T., Nishimura, S., and Miyazawa, T. (1997) Regional Polysterism in the GTP-Bound Form of the Human c-Ha-Ras Protein. *Biochemistry* **36**, 9109-9119

16. Scheidig, A. J., Burmester, C., and Goody, R. S. (1999) The pre-hydrolysis state of p21(ras) in complex with GTP: new insights into the role of water molecules in the GTP hydrolysis reaction of ras-like proteins. *Structure* **7**, 1311-1324

17. Cherfils, J., Menetrey, J., Le Bras, G., Janoueix-Lerosey, I., de Gunzburg, J., Garel, J. R., and Auzat, I. (1997) Crystal structures of the small G protein Rap2A in complex with its substrate GTP, with GDP and with GTPgammaS. *EMBO J.* **16**, 5582-5591

18. Merithew, E., Hatherly, S., Dumas, J. J., Lawe, D. C., Heller-Harrison, R., and Lambright, D. G. (2001) Structural plasticity of an invariant hydrophobic triad in the switch regions of Rab GTPases is a determinant of effector recognition. *J. Biol. Chem.* **276**, 13982-13988

19. Zhu, G., Liu, J., Terzyan, S., Zhai, P., Li, G., and Zhang, X. C. (2003) High resolution crystal structures of human Rab5a and five mutants with substitutions in the catalytically important phosphate-binding loop. *J. Biol. Chem.* **278**, 2452-2460

20. Schlichting, I., John, J., Frech, M., Chardin, P., Wittinghofer, A., Zimmermann, H., and Rosch, P. (1990) Proton NMR studies of transforming and nontransforming H-ras p21 mutants. *Biochemistry* **29**, 504-511

21. Reinstein, J., Schlichting, I., Frech, M., Goody, R. S., and Wittinghofer, A. (1991) p21 with a phenylalanine 28----leucine mutation reacts normally with the GTPase activating protein GAP but nevertheless has transforming properties. *J. Biol. Chem.* **266**, 17700-17706

22. Bendova, L., Jurecka, P., Hobza, P., and Vondrasek, J. (2007) Model of peptide bond-aromatic ring interaction: correlated ab initio quantum chemical study. *The journal of physical chemistry. B* **111**, 9975-9979

23. Choudhary, A., Newberry, R. W., and Raines, R. T. (2014) n-->pi* interactions engender chirality in carbonyl groups. *Organic letters* **16**, 3421-3423

24. Singh, S. K., and Das, A. (2015) The n --> pi* interaction: a rapidly emerging non-covalent interaction. *Physical chemistry chemical physics : PCCP* **17**, 9596-9612

25. Pauling, L., and Wilson, E. B. (1963) *Introduction to Quantum Mechanics*, Dover Publications, Inc., New York

26. Bishop, D. M. (1973) *Group Theory and Chemistry*, Dover Publications, Inc, New York

27. Gillespie, R. J., and Istvan Hargittai, I. (2012) *The VSEPR Model of Molecular Geometry*, Dover Publications
